# Local knowledge about sustainable harvesting and availability of wild medicinal plant species in Lemnos island, Greece

**DOI:** 10.1186/s13002-020-00390-4

**Published:** 2020-06-19

**Authors:** Dimitrios Papageorgiou, Penelope J. Bebeli, Maria Panitsa, Christoph Schunko

**Affiliations:** 1 Department of Sustainable Agricultural Systems, University of Natural Resources and Life Sciences Vienna (BOKU), Gregor-Mendel-Straße 33, 1180 Vienna, Austria; 2 Laboratory of Plant Breeding and Biometry, Department of Crop Science, Agricultural University of Athens, Iera Odos 75, 11855 Athens, Greece; 3 Division of Plant Biology, Department of Biology, University of Patras, 26504 Patras, Greece

**Keywords:** Ethnobotany, Ethnopharmacology, Folk medicine, Foraging, Near east, Plant conservation, Traditional medicine, Wild plant gathering

## Abstract

**Background:**

In Europe and the Mediterranean, over-exploitation and destructive harvesting techniques have been identified as two critical threats affecting the sustainable harvesting of wild medicinal plant (WMP) species. However, unsustainable harvesting is not an issue everywhere and localized assessments are needed. Local knowledge has been praised for its potential for local short-term assessments. In this study, we aimed to register the known, harvested, and locally utilized WMP species and understand local knowledge of harvesters about the ecological sustainability of WMP harvesting and the perceived changes of WMP availability.

**Materials and methods:**

This study was conducted on Lemnos island, Greece, in July and August 2018. Sixteen harvesters knowledgeable about gathering and using WMP were chosen through purposeful and snowball sampling. Successive free-lists provided insights on the taxa known, harvested, and utilized by harvesters and subsequent semi-structured interviews served to understand harvesting practices and perceived changes of WMP availability. Participant observation during seven harvesting walks allowed for additional insights and facilitated the collection of voucher specimens.

**Results:**

In total, 144 different plant taxa were listed as useful and 81 had been harvested in the prior 4 years. Medicinal applications were mainly related to digestive and respiratory system issues. A number of favorable harvesting practices suggested a high potential towards an ecologically sustainable harvest. Although, a decreased availability for certain plant taxa and harvesting sites was reported and mainly attributed to external factors such as pollution, unusually dry weather, intentional pastureland burning or chemicals in agriculture, but also destructive harvesting by less knowledgeable harvesters.

**Conclusions:**

Knowledgeable harvesters of Lemnos gather and use a considerable number of WMP taxa and possess local knowledge that supports an ecologically sustainable harvest. However, certain plant taxa and areas of the island were indicated to be under pressure from harvesting, unusual climatic conditions, and agricultural practices. Our approach confirmed that local knowledge should be taken into account for assessing the sustainability of WMP harvesting.

## Background

In Europe and the Mediterranean, over-exploitation and destructive harvesting techniques have been identified as two critical threats directly or indirectly affecting medicinal plant species [1, 2]. The main direct environmental consequence of unsustainable harvesting practices is the reduced reproduction, growth, and survival rates of the targeted species [3]. Such changes can consecutively destruct the ecosystem balances and influence the dynamics and structure of populations or even drive species to the brink of extinction [2–4].

However, unsustainable harvesting is not an issue everywhere [5] and, in contrary to that, most plant species have been found to be harvested sustainably [6]. Tolerance to the harvesting of wild plants varies and depends on several factors, including a plants’ lifespan, the part of the plant that is harvested, species abundance, the habitat where it is harvested, or species growth rate. For example, slow-growing plants are particularly susceptible to heavy harvesting, while those of weedy nature are less vulnerable [7, 8]. The assessment of ecological sustainability of harvesting thus needs to be based on the consideration of several factors together, most importantly the plant parts collected and its life form [9]. Estimating the sustainability of a harvested population and the effect of wild plant harvesting on other elements of the ecosystem requires long-lasting studies and can be hard to isolate and monitor.

Local people however many times rely on local knowledge to effectively and sustainably manage the harvest of wild plants [10–12]. Consequently, the involvement of local people in natural resource and harvesting monitoring regimes is considered key for its success [13, 14]. For short-term studies, research into the local knowledge about the sustainability of wild plant harvesting may be a promising approach to understand the sustainability of harvesting activities. For example, this includes the harvesting techniques and management practices, not only the specific methods used by the harvesters prior, during, or after harvesting, but also observations of plant populations and harvesting activities of other harvesters. It may also act as a tool in detecting early signs of changes in species and population trends and create the groundwork for developing scientific monitoring for conservation [11].

In this study, we built on these insights and make use of local knowledge for assessing the sustainability of harvesting activities. We aimed to (a) register the known, harvested, and locally utilized wild medicinal plant (WMP) species, (b) understand the local knowledge of harvesters about an ecologically sustainable WMP harvesting, and (c) understand perceived changes of harvesters of WMP availability. We thereby regard local knowledge as epistemologically distinct from scientific knowledge and being valuable on its own rather than searching for its validation with scientific methods [15].

## Methods

### Study area

**Fig. 1:**
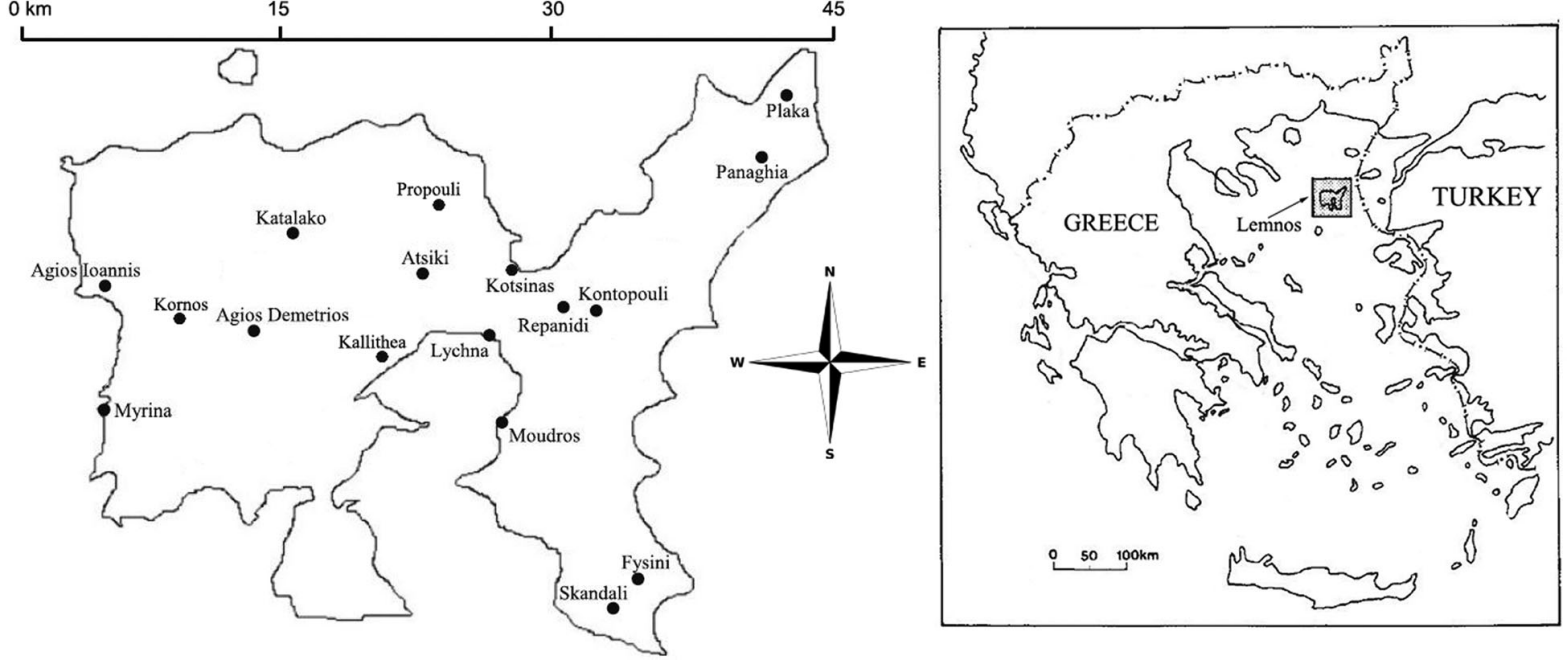
Map and location of Lemnos island. Village names indicate places where interviews and/or harvesting walks were conducted (figure adapted from [17, 18])

The study was conducted on Lemnos island—in contemporary sources also spelled Limnos—Greece, occupying about 482 km^2^ and biogeographically belonging to the North Aegean Sea [16, 17] (Fig. 1). It resides in the Prefecture of Lesvos (39° 46′–40° 02′ N, 25° 02′–25° 26′ E) [16, 18] and the climate of the area is the Mediterranean with mild winters, dry hot summers, and mean annual precipitation of about 500 mm [19, 20].

From an ecological perspective, the island is characterized by a variety of habitat types including flat coastlines, lagoons, wetlands, marshes, sand dunes, remnants of a Valonia oak (*Quercus ithaburensis* Decne. subsp. *macrolepis* (Kotschy) Hedge & Yalt.) forest, agricultural crops, and extended phrygana [16, 18]. Despite its diverse environments, the flora of the island is rather poor compared to neighboring islands, with regard to the number of plant species, but still maintains significant ecological value due to its multifarious vegetation formations [17].

Despite its high ecological and cultural value landscapes, the island’s natural ecosystems have been greatly degraded [18, 21]. The smooth topography with medium inclinations allowed intense human activities to take place on almost the entire island. Extended agriculture, tourism facilities, and the traditional agropastoral practice of burning and intense grazing (sheep and goats) have drastically affected the formation of natural ecosystems and their vegetative composition [18].

Nationwide restrictions for gathering WMP species apply in Lemnos. Current Greek legislation allows the extraction of two kilos of fresh plant material per plant species and person. Uprooting, underground plant part removal, and orchid harvesting are completely forbidden [22]. For the prefecture of Lesvos, harvesting limits for thyme (*Thymus* spp.), pennyroyal (*Mentha* sp.), wild mint (*Mentha* sp.), oregano (*Origanum* spp.), mountain tea (*Sideritis* spp.), Saint John’s wort (*Hypericum* spp.), and sage (*Salvia* spp.) are set to half a kilo of fresh plant material per plant species and person per day. In cases of larger quantities, permission needs to be acquired from the local forestry authorities. Several medicinal plant species are fully protected and their harvesting is prohibited in the prefecture of Lesvos [23].

### Sampling strategy

The sampling strategy contained purposeful and snowball sampling of knowledgeable WMP harvesters living on the island of Lemnos [24]. The geographic coverage of the island was set as a secondary priority due to its diverse habitats, whereas no limitations on socio-demographic characteristics such as age, marital status, income level, education level, or duration of residence on the island were set.

During two field trips, knowledgeable WMP harvesters were located by asking local people or reaching out through the social media Greek group “Friends of old metropolis of Lemnos” with a large number of members and close relevance to Lemnos’ cultural, environmental, and farming issues. The sample development stopped at 16 informants (nine female and seven male) since a saturation point had been reached, as there was very little new information coming out from the last five interviews and no more knowledgeable WMP harvesters could be identified. Respondents’ ages varied from 32 to 78 years, with an arithmetic mean of 57 years. Fifteen of the respondents were permanent residents on the island, with periods of residency from five to 72 years, and an arithmetic mean of 23 years, while one was a regular summer-visitor for the last 50 years and permanent resident prior to that. Twenty-five percent of the informants were non-natives, meaning that they have no ancestral connection with the island of Lemnos.

### Data collection

During July and August 2018, sixteen face-to-face interviews were conducted using semi-structured interviews and successive free lists [24, 25]. Participant observation was conducted with six informants during seven harvesting walks [24]. Interviews took place at the town of Myrina and the villages; Agios Demetrios, Lychna, Agios Ioannis, Atsiki, Fysini, Skandali, Kallithea, Kontopouli, Panaghia, and Moudros.

#### Semi-structured interviews and successive free listing

An interview guide with a list of overarching topics and subsequent questions was used to structure the semi-structured interviews (Additional file 1). After being given a short introduction to the research topic, respondents were guided to talk about the availability and development of their knowledge about WMP harvesting, selection of harvesting sites, harvesting practices, and environmental awareness.

Informants were then asked to list all of the wild-growing medicinal plant species of Lemnos they know, using the free-listing question: “Μπορείς σε παρακαλώ να μου πεις όλα τα βότανα/φαρμακευτικά φυτά που γνωρίζεις ότι βγαίνουν άγρια, άρα από μόνα τους, στο νησί;” (literal translation: “Can you please list all the medicinal plants you know that grow wild, thus on their own, on the island?”). When the interviewees could not recall more plant names, they were asked to think about the plant species growing in the different times of the year. When they ran out of ideas again, they were asked to think about the different sites where medicinal plants were growing. In the end, the plants already listed were slowly repeated and informants were asked whether any other wild-growing medicinal plants came to their mind.

All informants were then asked to point out which of the previously indicated plants they harvested by themselves in Lemnos at least once within the last 4 years. If a plant was harvested, respondents were asked to mention the plant parts harvested, harvesting time, equipment used, and ways of processing, preparation, and use. The answers to the successive free lists were written down in a structured questionnaire.

#### Participant observation and wild medicinal plant voucher specimens

Participant observation during harvesting walks was used to develop deeper insights on respondents’ knowledge about WMP harvesting. The intention was to investigate behaviors, thoughts, and actions that people might not have been able to explain in the interviews [26]. Harvesters were asked to point out, name, and collect all WMPs they saw during the walk.

Nine harvesting walks were conducted in total at nine different sites, most of them with interviewed knowledgeable harvesters that expressed willingness to join on a walk to identify and harvest WMPs. Field notes were taken in the Greek language throughout the walks and brain protocols were completed afterwards.

Harvesting sites were identified by harvesters as possible locations to find a big variety of WMPs mentioned in their interviews. The sites were in the areas of Katalakko, Kotsinas, Lychna-Repanidi, Moudros, Plaka, Propouli, and Therma (near Kornos) (Fig. 1). During the harvesting walks, 83 WMP voucher specimens were collected and deposited at the UPA Herbarium (Department of Biology, University of Patras, Greece).

### Data analysis

#### Semi-structured interviews, successive free listing, and participant observation

Semi-structured interviews were analyzed using qualitative content analysis [27]. Therefore, selected sections of the voice recordings, that were identified as relevant to answer the research questions, were transcribed in the Greek language. Then, deductive coding was applied, whereas initial codes were derived from points of interest arising from the research questions and complemented with additional inductive codes that came up during the coding process [24, 27]. The qualitative data analysis software QDA Miner Lite was used for coding [28]. The coded content was translated to English, indexed in Microsoft Excel spreadsheets [29], and summarized code-by-code following the steps of paraphrasing, generalization, and reduction [27]. Field notes resulting from participant observation were translated to English and included in the qualitative content analysis.

Data resulting from successive free-listing were digitalized in Microsoft Excel spreadsheets [29] and analyzed with descriptive statistics by calculating arithmetic means and sums. Greek plant vernacular names were assigned to their phonetic attribution according to the International Phonetic Alphabet [30].

#### Wild medicinal plant voucher specimens

The 83 voucher specimens that were identified with their vernacular names by the participants, corresponded to 63 different plant taxa. Nomenclature follows ‘The Plant List’ [31] except for the species of *Stachys cretica* subsp. *lesbiaca* Rech. Fil. (that is not found in ‘The Plant List’) and *Crithmum maritimum* L. (an unresolved case in ‘The Plant List’). These two taxa follow Strid [32] and Dimopoulos et al. [33, 34]. In most cases, the correspondence between a vernacular and scientific name is on a one-to-one basis. However, there are instances where a plant vernacular name is assigned to more than one plant species, sometimes even belonging to different botanical genera and families (Table 1).

**Table 1:** List of wild medicinal plants known by knowledgeable harvesters of Lemnos (*n* = 16)

Scientific name	Family	v	f	Vernacular name in Greek	Phonetic attribution
*Thymbra capitata* (L.) Cav. ^a^	*Lamiaceae*	DP108	16	Θυμάρι	Thymári
*Mentha pulegium* L. ^a^	*Lamiaceae*	DP144	15	Φλισκούνι, Φιλσκούνι, Βλισκούνη, Βιλσκούν, Άγρια Μέντα	Fliskúni, Fiʎskúni, Vliskúni, Viʎskún, Άɣria Ménda
*Matricaria chamomilla* L. ^a^	*Compositae*	Obs.	14	Χαμομήλι	Xamomíli
*Origanum vulgare* L. subsp. *hirtum* (Link) Ietsw. ^a^	*Lamiaceae*	DP146	14	Ρίγανη	Ríɣani
*Hypericum perfoliatum* L. and *Hypericum perforatum* L. ^a^	*Hypericaceae*	DP107, DP336	14	Βαλσαμόχορτο, Σπαθόχορτο, Σπαθοβότανο	Valsamóxorto, Spathóxorto, Spathovótano
*Malva sylvestris* L. ^a^	*Malvaceae*	DP132	12	Μολόχα	Molóxa
*Salvia spp.*^*b*^	*Lamiaceae*	DP115	11	Φασκομηλιά, Φασκόμηλο, Τσάι του βουνού	Faskomiʎá, Faskómilo, Tsái tu vunú
*Taraxacum* spp.	*Compositae*	DP167	9	Πικροράδικο, Ταραξάκο, Αγριομάρουλο, Ραδίκι Ταραξάκο, Πικραλίδα	Pikroráðiko, Taraksáko, Aɣriomárulo, Raðíki Taraksáko, Pikralíða
*Urtica* sp.	*Urticaceae*	Obs.	9	Τσουκνίδα	Tsukníða
*Sonchus oleraceus (*L.) L.	*Compositae*	DP161	8	Ζοχοί, Ζοχός, Ζοχάρια	Zoçí, Zoxós, Zoxárʝa
*Cynodon dactylon* (L.) Pers.	*Poaceae*	DP170	8	Αγριά, Αγριάδα	Aɣriá, Aɣriáða
*Rosa canina* L. ^a^	*Rosaceae*	DP137, DP173	8	Αγριοτριανταφυλλιά, Άγρια Τριανταφυλλιά	Aɣriotriandafyʎá, Άɣria triandafyʎá
*Crithmum maritimum* L.	*Apiaceae*	DP131	7	Κρίταμα, Κρίταμο	Krítama, Krítamo
*Asparagus acutifolius* L. ^a^	*Asparagaceae*	DP150	7	Άγρια Σπαράγγια, Άγριο Σπαράγγι, Αγριοσπαραγγιά, Σπαράγγι, Σπαράγγια	Άɣria Sparaɟá, Άɣriο Sparáɟi, Άɣriosparaɟá, Sparáɟi, Sparaɟiá
*Hypericum triquetrifolium* Turra	*Hypericaceae*	DP135	7	Αγούδουρας	Aɣúðuras
*Foeniculum vulgare* Mill. ^*a b*^	*Apiaceae*	DP141	6	Άγριος Μάραθος, Μάραθος, Άγριος Άνηθος	Άɣrios Márathos, Márathos, Άɣrios Άnithos
*Tordylium apulum* L. ^a^	*Apiaceae*	DP196	6	Καυκαλήθρες, Καυκαλήθρα	Kafkalíthres, Kafkalíthra
*Silybum marianum* (L.) Gaertn. ^a^	*Compositae*	Obs.	7	Γαϊδαράγκαθο, Γαϊδουράγκαθο, Γαϊδουράγκαθο το καθεαυτού, Σίλυβο	ɣai̯ðurágkatho, ɣai̯ðarágkatho, ɣai̯ðurágkatho to katheaftú, Sílyvo
*Capparis spinosa* L. ^a^	*Capparaceae*	DP126	6	Κάπαρη	Kápari
*Portulaca oleracea* L.	*Portulacaceae*	DP160	6	Γλιστρίδα, Αντράκλα	ɣlistríða, Andrákla
*Dittrichia viscosa* (L.) Greuter	*Compositae*	DP103	5	Ακόνιζα, Ακόντζα, Ακόνιζα μικρή, Ακόνιζα μεγάλη	Akóniza, Akondzá, Akóniza mikrí, Akóniza meɣáli
*Sinapis arvensis* L. subsp. *arvensis*	*Brassicaceae*	Obs.	5	Βρούβες, Γρούβα, Γρούβες, Τσιμπητά	Vrúves, ɣrúves, Tsimbitá
*Opuntia ficus-indica* (L.) Mill. ^*b*^	*Cactaceae*	DP176	5	Άγρια Φραγκοσυκιά, Φραγκοσυκιά	Άɣria Fragosycá, Fragosycá
*Mentha aquatica* L.	*Lamiaceae*	Obs.	5	Άγρια Μέντα, άγριος Δυόσμος	Άɣria Ménda, Άɣrios ðʝósmos
*Teucrium capitatum L.*	*Lamiaceae*	DP101, DP118	5	Στομαχοβότανο, Παναγιόχορτο, Λαγοκοιμηθιά, Βοτάνι της Παναγίας, Της Παναγιάς το χόρτο	Stomaxovótano, Panaʝóxorto, Laɣokimithçá, Votáni tis Panaʝías, Tis Panaʝás to xórto
*Pyrus communis* L.	*Rosaceae*	DP152	5	Αγριοαχλαδιά, Άγριο Αχλάδι, Γκορτσιά	Aɣrioxlaðʝá, Άɣrio Axláði, Gortsçá
*Rubus sanctus* Schreb. ^a^	*Rosaceae*	DP138	5	Άγριος Βάτος, Βατομουριά, Άγρια Βατομουριά, Βατόμουρο	Άɣrios Vátos, Vatomurʝá, Άɣria Vatomurʝá, Vatómuro
*Anthriscus* sp.	*Apiaceae*	Obs.	4	Μυρώνι, Μυρώνια	Myróni, Myrónia
*Calendula arvensis* M.Bieb. ^a^	*Compositae*	Obs.	4	Άγρια Καλέντουλα	Άɣria Kaléndula
*Crepis zacintha* (L.) Babc.	*Compositae*	DP188	4	Βότανο για τις μυρμηγκιές, Φυτό για μυρμηγκιές, Αστεροειδής, Χόρτο για ορνιθόκωλους	Vótano ʝa tis myrmiɟés, Fitó ʝa myrmiɟés, Asteroi̯ðís, Xórto ʝa ornithókolus
*Scolymus hispanicus* L.	*Compositae*	Obs.	4	Σκουμπρουγούλι, Σκομπρογούλια, Σκόμπρος, Γαϊδουράγκαθο	Skubruɣúli, Skobroɣúʎia, Skóbros, ɣai̯ðurágkatho
*Eruca vesicaria* (L.) Cav.	*Brassicaceae*	DP134	4	Άγρια Ρόκα	Άɣria Róka
*Cistus* sp.	*Cistaceae*	DP102	4	Κίστος, Λαδανιά, Χαμοκίσαρο	Cístos, Laðaɲá, Xamocísaro
*Ecballium elaterium* (L.) A. Rich.	*Cucurbitaceae*	DP157	4	Άγριο αγγούρι, Πικράγγουρα, Πικράγγουρο, Πικραγγουριά	Άɣrio Aggúri, Pikrággura, Pikrágguro, Pikraggurʝá
*Quercus coccifera* L.	*Fagaceae*	DP186	4	Πουρνάρι, Δρυς, Βελανιδιά	Purnári, ðRýS, Velaniðiá
*Melissa officinalis* L. ^a^	*Lamiaceae*	DP140	4	Άγριο Μελισσόχορτο, Μελισσόχορτο	Άɣrio Melisóxorto, Melisóxorto
*Mentha spicata* L. ^a^	*Lamiaceae*	Obs.	4	Αγριοδυόσμος, Άγριος Δυόσμος	Aɣrioðʝósmos, Άɣrios ðʝósmos
*Rosmarinus officinalis* L. ^*a b*^	*Lamiaceae*	DP179	4	Δενδρολίβανο, Δενδρολίβανος, Δενδρολίβανος αυτοφυής	ðenðrolívano, ðenðrolívanos, ðenðrolívanos aftofyi̯s
*Papaver dubium* L.	*Papaveraceae*	DP189	4	Παπαρούνα rhoeas, Παπαρούνες, Κοτσνάδες	Paparúna rhoéas, Paparúnes, Kotsnáðes
*Plantago weldenii* Rchb.	*Plantaginaceae*	DP158	4	Πετινός, Πετιναρέλι, Πετναρούδ, Πετιναράκι	Petinós, Petinaréli, Petnarúð, Petinaráci
*Rumex crispus* L. ^a^	*Polygonaceae*	DP193	4	Λάπατα, Άγριο Λάπαθο, Λάπαθο	Lápata, Άɣrio Lápatho, Lápatho
*Rumex obtusifolius* L.	*Polygonaceae*	DP183	4	Σεύκλο,Σεύκλα, Άγρια Σέσκουλα, Σέσκουλα, Μικρό Λάπαθο	Séfklo, Séfkla, Άɣrio Séfklo, Séskula, Mikró Lápatho
Verbascum lasianthum Boiss. ex Benth. and *Euphorbia characias* L. and *Euphorbia seguieriana* Neck.	*Scrophulariaceae, Euphorbiaceae, Euphorbiaceae*	DP116, DP124, DP162	4	Φλόμος	Flómos
*Datura stramonium* L. ^a^	*Solanaceae*	Obs.	4	Ντάντουρας, Ντατούρα, Άγριο Διαβολόχορτο, Διαβολόχορτο	Dáduras, Datúra, Άɣrio ðʝavolóxorto, ðʝavolóxorto
*Tribulus terrestris* L. ^a^	*Zygophyllaceae*	DP106, DP172	4	Αντρίβολας, Τριβόλι	Adrívolas, Trivóli
*Daucus carota* L.	*Apiaceae*	DP163	3	Άγριο Καρότο, Τραχανόχορτο	Άɣrio Karóto, Traxanóxorto
*Lactuca serriola* L.	*Compositae*	Obs.	3	Αγριομάρουλο	Aɣriomárulo
*Cichorium intybus* L. and *Cichorium pumilum* Jacq.	*Compositae*	DP125, DP166	3	Κιχώριο, Ραδίκι, Ραδίκι με μπλε άνθος	Cixório, Raðíki, Raðíki me ble ánthos
*Cichorium* spp. *and Taraxacum* spp.	*Compositae*	DP125, DP166, DP167	3	Ραδίκια	Raðíkia
*Raphanus raphanistrum* L. ^a^	*Brassicaceae*	DP159	3	Άγρια Ρεπανίδα, Ρεπανίδα, Ρεπανίδες	Άɣria Repaníða, Repaníða, Repaníðes
*Cardamine hirsuta* L.	*Brassicaceae*	Obs.	3	Αγριοκάρδαμο, Άγριο Kάρδαμο	Aɣriokárðamo, Άɣrio Kárðamo
*Ephedra foeminea* Forssk. ^a^	*Ephedraceae*	Obs.	3	Πολυκόμπι, Πολύκομπος	Polykómbi, Políkombos
*Crocus* sp.	*Iridaceae*	Obs.	3	Κρόκος	Krókos
*Althaea officinalis* L. ^b^	*Malvaceae*	DP104	3	Δενδρομολόχα, Αλθέα	ðenðromolóxa, Althéa
*Ficus carica* L. ^a b^	*Moraceae*	DP119, DP120, DP121	3	Συκιά	Sycá
*Plantago lanceolata* L.	*Plantaginaceae*	DP164	3	Πεντάνευρο	Pendánevro
*Galium aparine* L. and *Polypogon monspeliensis* (L.) Desf.	*Rubiaceae, Poaceae*	Obs., DP130	3	Γάλιο, Κολλτσίδα, Κολλιτσίδα	ɣálio, Koʎtsíða, Kolitsíða
*Solanum nigrum* L.	*Solanaceae*	DP155	3	Αγριοντοματιά, Αγριοντοματούδι, Στίφνος	Αɣriοντοματçá, Αɣriondomatúði, Stífnos
*Vitex agnus-castus* L.	*Lamiaceae*	DP111	3	Λιγαριά	Liɣarʝá
*Allium* sp.	*Amaryllidaceae*	Obs.	2	Αγριόσκορδο	Aɣriόskorðo
*Amaranthus retroflexus* L. ^a^	*Amaranthaceae*	DP154	2	Βλήτα	Vlíta
*Apium* sp.	*Apiaceae*	Obs.	2	Αγριοσέλινο, Άγριο Σέλινο	Aɣriosélino, Aɣrio Sélino
*Helichrysum stoechas* (L.) Moench ^a^	*Compositae*	Obs.	2	Ελίχρυσος	Elíxrysos
*Cichorium* sp.	*Compositae*	Obs.	2	Σταμναγκάθι	Stamnagáthi
*Alkanna tinctoria* Tausch ^a^	*Boraginaceae*	DP128, DP129	2	Αλκάνα, Alkanna tinctoria	Alkána, Alkanna tinctoria
*Echium plantagineum* L.	*Boraginaceae*	Obs.	2	Βοϊδόγλωσσα	Voi̯ðóɣlosa
*Sinapis alba* L.	*Brassicaceae*	Obs.	2	Λαψάνες, Βρούβες	Lapsánes, Vrúves
*Cuscuta* sp. and *Orobanche* sp.	*Convolvulaceae, Orobanchaceae*	DP109, DP194	2	Λύκος	Lýkos
*Robinia pseudoacacia* L. ^c^	*Leguminosae*	Obs.	2	Ακακία	Akakía
*Ballota acetabulosa* (L.) Benth.	*Lamiaceae*	DP174	2	Φυτιλιά, Φυτιλάκι, Φυτιλάκια	Fytiʎá, Fytiláci, Fytiláca
*Olea europaea* L. subsp. *oleaster* (Hoffmanns. & Link) Negodi ^c^	*Oleaceae*	Obs.	2	Αγριοελιά, Αγριελιά	Aɣrioeʎá, Aɣrieʎá
*Limonium sinuatum* (L.) Mill.	*Plumbaginaceae*	DP117	2	Προβάτσες, Αμάραντα	Provátses, Amáranda
*Cydonia* sp.	*Rosaceae*	Obs.	2	Άγρια Κυδωνιά	Άɣria Cyðoɲá
*Sarcopoterium spinosum* (L.) Spach	*Rosaceae*	Obs.	2	Αστοιβιά, Αστοιβή	Astivʝá, Astiví
*Ruta graveolens* L.	*Rutaceae*	DP168, DP182	2	Απήγανος	Apíɣanos
*Salix alba* L. ^a^	*Salicaceae*	Obs.	2	Ιτιά	Itçá
*Mandragora* sp.	*Solanaceae*	Obs.	2	Μανδραγόρας	Manðraɣóras
*Ulmus minor* Mill.	*Ulmaceae*	DP139	2	Καραγάτσι, Φτελιά	Karaɣátsi, Fteʎá
*Parietaria judaica* L. ^a^	*Urticaceae*	DP148	2	Περδικάκι	Peðikáci
*Acanthus spinosus* L.	*Acanthaceae*	Obs.	1	Άνγκαθος	Άngkathos
*Allium* sp.	*Amaryllidaceae*	Obs.	1	Άγριο πράσσο	Άɣrio Prásso
*Allium* sp.	*Amaryllidaceae*	Obs.	1	Αγριοκρέμμιδο	Aɣriokrémiðo
*Amaranthus* sp.	*Amaranthaceae*	Obs., DP112	1	Αμάρανθος	Amáranthos
*Salicornia europaea* L.	*Amaranthaceae*	Obs.	1	Σαλικόρνια	Salikórnia
*Petroselinum Crispum* (Mill.) Fuss	*Apiaceae*	Obs.	1	Αγριομαιδανός	Aɣriomai̯ðanós
*Hedera helix* L.	*Araliaceae*	Obs.	1	Κισσός	Cissós
*Asphodelus ramosus* L. subsp. *ramosus*	*Xanthorrhoeaceae*	Obs.	1	Ασπόρδουλας	Aspórðulas
*Cynara cardunculus* L.	*Compositae*	Obs.	1	Άγρια Αγκινάρα	Άɣria Agkinára
*Centaurea* sp.	*Compositae*	Obs.	1	Κενταύριο	Kendávrio
*Centaurea benedicta* (L.) L.	*Compositae*	Obs.	1	Κνίκος	Kníkos
*Anthemis* sp.	*Compositae*	Obs.	1	Μαργαρίτες	Marɣarítes
*Carthamus dentatus* subsp. *ruber* (Link) Hanelt	*Compositae*	DP105	1	Του Χριστού το αγκαθάκι	Tu Xristú to agatháci
*Cardopatium corymbosum* (L.) Pers.	*Compositae*	Obs.	1	Χαμολιός	xamoʎiós
*Borago officinalis* L.	*Boraginaceae*	Obs.	1	Μπουράτζα	Burádza
*Sambucus nigra* L. ^a^	*Adoxaceae*	DP122	1	Σαμπούκος	Sabúkos
*Saponaria officinalis* L.	*Caryophyllaceae*	Obs.	1	Σαπουνόχορτο	Sapunóxorto
*Convolvulus arvensis* L.	*Convolvulaceae*	DP133	1	Μπαρμποκλάδα, Περικοκλάδα	Barbokláða, Perikokláða
*Cupressus* sp. ^a c^	*Cupressaceae*	Obs.	1	Κυπαρίσσι	Cyparísi
*Dioscorea communis* (L.) Caddick & Wilkin ^a^	*Dioscoreaceae*	Obs.	1	Οβριές, Αβρονιές	Ovriés, Avroɲiés
*Vicia villosa* Roth	*Leguminosae*	Obs.	1	Αγριοβίκος, Καβαλαριά	Aɣriovíkos, Kavalarʝá
*Trifolium* sp.	*Leguminosae*	Obs.	1	Αγριοτριφύλλι	Aɣritrifýli
*Spartium junceum* L.	*Leguminosae*	Obs.	1	Σπάρτα	Spárta
*Centaurium pulchellum* (Sw.) Druce	*Gentianaceae*	DP149	1	Βότανο για διάρροια	Vótano ʝa ðiária
*Erodium cicutarium* (L.) L'Her. ^a^	*Geraniaceae*	Obs.	1	Της πέρδικας το νύχι	Tis pérðikas to nýçi
*Hypericum olympicum* L.	*Hypericaceae*	DP123	1	Χελωνόχορτο	çelonóxorto
*Juglans regia* L. ^c^	*Juglandaceae*	Obs.	1	Καρυδιά	Karyðʝá
*Stachys cretica* subsp. *lesbiaca* Rech. Fil.	*Lamiaceae*	DP184	1	Ασφακιά	Asfacá
*Thymus* sp.	*Lamiaceae*	Obs.	1	Θρούμπι	Thrúbi
*Origanum majorana* L.	*Lamiaceae*	Obs.	1	Ματζουράνα	Madzurána
*Sideritis* sp.	*Lamiaceae*	Obs.	1	Σιδερίτης	Siðerítis
*Laurus nobilis* L.	*Lauraceae*	Obs.	1	Δάφνη	ðáfni
*Morus* sp.	*Moraceae*	Obs.	1	Σκάμνια	Skámɲa
*Eucalyptus globulus* Labill. ^c^	*Myrtaceae*	Obs.	1	Ευκάλυπτος	Efkályptos
*Ophrys* sp.	*Orchidaceae*	Obs.	1	Ορχιδέα (σαλέπι)	Orxiðéa ʝa salépi
*Glaucium flavum* Crantz	*Papaveraceae*	Obs.	1	Κίτρινη Παπαρούνα	Cítrini Paparúna
*Papaver somniferum* L.	*Papaveraceae*	Obs.	1	Παπαρούνα οπιούχος	Paparúna opiúxos
*Phytolacca americana* L.	*Phytolaccaceae*	DP156, DP157	1	Φυτόλακκα	Fytólaka
*Pinus brutia* Ten. ^c^	*Pinaceae*	Obs.	1	Πεύκο	Péfko
*Platanus orientalis* L. ^a^	*Platanaceae*	Obs.	1	Πλατάνι	Platáni
*Avena sterilis* L.	*Poaceae*	Obs.	1	Αγριοβρώμη	Aɣriovrómi
*Rumex* sp.	*Polygonaceae*	Obs.	1	Νερολάπαθα	Nerolápatho
*Anemone* sp.	*Ranunculaceae*	Obs.	1	Ανεμώνες	Anemónes
*Crataegus azarolus* L.	*Rosaceae*	DP141, DP187	1	Κράτεγος, Τρικοκιά	Kráteɣos, Trikociá
*Prunus dulcis* (Mill.) D.A.Webb var.	*Rosaceae*	Obs.	2	Άγρια Αμυγδαλιά, Πικραμύγδαλο	Άɣria Amyɣðaʎá, Pikramíɣðalo
*Prunus spinosa* L.	*Rosaceae*	DP191	1	Προύνες, Άγρια Μούσκλα	Prúnes, Άɣria Múskla
*Verbascum lasianthum* Boiss. ex Benth.	*Scrophulariaceae*	DP116	1	Αγριμόνιο	Aɣrimónio
*Verbascum* sp.	*Scrophulariaceae*	Obs.	1	Βερμπάσκο, Πλόνος	Verbásko, Plónos
*Ailanthus altissima* (Mill.) Swingle	*Simaroubaceae*	Obs.	1	Ασλάνιδες	Aslániðes
*Solanum villosum* Mill.	*Solanaceae*	Obs.	1	Άγριο ντοματάκι	Άɣrio ndomatáci
*Hyoscyamus albus* L. ^a^	*Solanaceae*	DP153, DP181	1	Υοσκύαμος	Yi̯oskíamos
*Tilia* sp. ^c^	*Tiliaceae*	Obs.	1	Τίλιο or Φλαμουριά	Tíʎo, Flamurʝá
*Misopates orontium* (L.) Raf.	*Plantaginaceae*	DP169	1	Φτόσμος	Ftósmos
*Viola kitaibeliana* Schult.	*Violaceae*	Obs.	1	Άγρια Βιολέτα	Άɣria Vʝoléta

Abbreviations: *f* frequency of plant referrals in free-listing exercise, *v* voucher specimen number, *Obs*. observation

^a^Plant species presented in the study of Axiotis et al. [45] as being utilized for medicinal purposes by locals in the Greek islands of North Aegean Region

^b^Plant taxa also growing in people’s gardens or agricultural land as cultivated

^c^Plant taxa cited as exclusively cultivated in people’s gardens or agricultural land

The vernacular plant names, which were mentioned during the interviews but did not correspond to voucher specimens are linked to 82 different plant taxa using identification information obtained from the literature [35]. Due to the lack of identification data for 21 of the cited vernacular names, these plants were not included in the results.

## Results

### Wild medicinal plants of Lemnos

#### Plants known

Our respondents explained that knowledge on harvesting and utilizing wild plants was necessary for the past as Lemnos’ residents were principally dependent on the island’s provisions and thus had to manage these resources in a sustainable way. Nowadays, this body of local knowledge on WMP harvesting was reported to be comparably small and only few individuals having considerable knowledge.

Respondents cited 439 plant items altogether, which corresponded to 144 different plant taxa belonging to 60 different plant families (Table 1). Each interviewee listed between 10 and 67 plant items with an arithmetic mean of 27 responses per person.

**Fig. 2:**
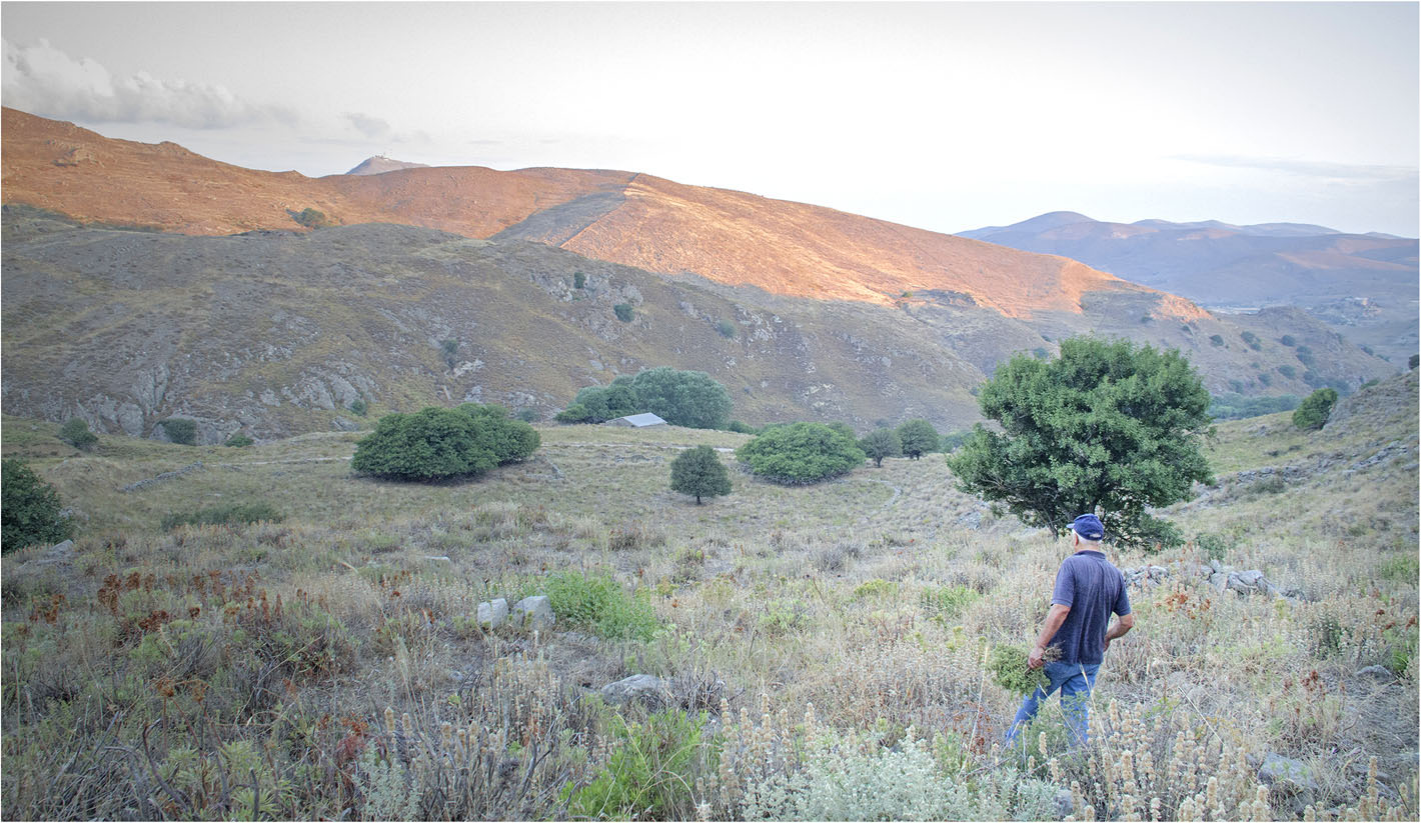
Local harvester collecting oregano (O. *vulgare* L. subsp. *hirtum*), one of the most widely known and harvested plants in Lemnos

The most frequently listed plant taxa are *Thymbra capitata* (L.) Cav., *Origanum vulgare* subsp. *hirtum* (Link) letsw. (Fig. 2), *Hypericum perfoliatum* L., *Hypericum perforatum* L., *Mentha pulegium* L., *Matricaria chamomilla* L., *Malva sylvestris* L., *Cynodon dactylon* (L.) Pers., *Rosa canina* L., and *Sonchus oleraceus* (L.) L. Each of these plant taxa was listed by at least 50% of the respondents. Species belonging to the plant genera of *Salvia*, *Taraxacum*, and *Urtica* were also listed by more than 50% of the respondents.

Although asked about medicinal plants growing in the wild, six of the plant taxa mentioned as gathered in the wild were also reported to be cultivated in homegardens and within or around agricultural land. These are *Althaea officinalis* L., *Rosmarinus officinalis* L., *Foeniculum vulgare* Mill., *Opuntia ficus indica* (L.) Mill., *Ficus carica* L., and *Salvia* sp. Taxa like *Robinia pseudoacacia* L., *Olea europaea* L. subsp. *oleaster* (Hoffmanns. & Link) Negodi, *Juglans regia* L., *Eucalyptus globulus* Labill., *Pinus brutia* Ten., *Cupressus* spp., and *Tilia* spp. mentioned as wild-gathered are only planted on the island (Table 1).

#### Plants harvested

Among the 144 plant taxa known by respondents, 81 had been harvested by the respondents at least once in the last 4 years (Table 2). These 81 plant taxa belong to 38 different plant families. The most commonly harvested plant taxa, collected by at least half of the respondents, are *T. capitata*, *O. vulgare* subsp*. hirtum*, *H. perfoliatum*, *H. perforatum*, *Mentha pulegium*, *Matricaria chamomilla*, and *Malva sylvestris.* Plant taxa belonging to the family of *Compositae* were most frequently reported as gathered (14 taxa), followed by the families of *Lamiaceae* (nine taxa), *Apiaceae*, and *Rosaceae* (six taxa each).

**Table 2:** List of wild medicinal plants collected by knowledgeable harvesters of Lemnos (*n* = 16)

Scientific name	Family	f^**a**^	Harvested part(s) ^**b**^	Harvesting month(s)^**c**^	Harvesting equipment^**d**^
*Thymbra capitata* (L.) Cav.	*Lamiaceae*	15	46% Flower, 54% upper stem	June-July (April-August)	79% Gardening scissor, 7% knife, 14% hand
*Origanum vulgare* L. subsp. *hirtum* (Link) letsw.	*Lamiaceae*	12	Upper stem	July (June-August)	43% Gardening scissor, 20% knife, 14% scythe, 23% hand
*Hypericum perfoliatum* L. and *Hypericum perforatum* L.	*Hypericaceae*	11	22% Flower, 78% upper stem	May-June (March-July)	22% Gardening scissor, 78% Hand
*Matricaria chamomilla* L.	*Compositae*	10	Flower	May (April-June)	8% Gardening scissor, 92% Hand
*Mentha pulegium* L.	*Lamiaceae*	10	Upper stem	July (April-November)	25% Gardening scissor, 22% knife–53% hand
*Malva sylvestris* L.	*Malvaceae*	8	83% Flower, 17% upper stem	April May (March-July)	Hand
*Salvia spp.*	*Lamiaceae*	7	57% Upper stem, 43% flower	June-September (all year)	41% Gardening scissor, 33% knife, 36% hand
*Taraxacum* spp.	*Compositae*	7	Whole aerial	November-March (October-July)	89% Knife, 11% hand
*Crithmum maritimum* L.	*Apiaceae*	6	38% Leaves, 62% upper stem	June (March-September)	29% Gardening scissor, 71% hand
*Portulaca oleracea* L.	*Portulacaceae*	6	20% Leaves, 80% upper stem	June-July (March-September)	20% Gardening scissor, 20% knife, 60% hand
*Sonchus oleraceus* (L.) L.	*Compositae*	6	Whole aerial (young/fresh leaves)	November-April (October-May)	88% Knife, 12% hand
*Rosa canina* L.	*Rosaceae*	5	29% Flower, 71% fruit	May (March-July) & September-October (November)	12% Gardening scissor, 88% hand
*Tordylium apulum* L.	*Apiaceae*	5	Whole aerial (young/fresh leaves)	February-March (October-March)	83% Knife, 17% hand
*Asparagus acutifolius* L.	*Asparagaceae*	4	40% Whοle aerial, 60% upper stem	March (November-May)	Hand
*Capparis spinosa* L.	*Capparaceae*	4	Upper stem—including flower buds, fruit and leaves	May-June (April-July)	25% Gardening scissor, 75% hand
*Foeniculum vulgare* Mill	*Apiaceae*	4	20% Upper stem, 10% flower, 40% leaves, 30% feed	March-September (all year)	Hand
*Hypericum triquetrifolium* Turra	*Hypericaceae*	4	25% Flower, 75% upper stem	June (May-July)	20% Gardening scissor, 80% hand
*Cichorium intybus* L. and *Cichorium pumilum* Jacq.	*Compositae*	3	Whole aerial (young/fresh leaves)	October-December (October-April)	Knife
*Cichorium* spp. and *Taraxacum* spp.	*Compositae*	3	Whole aerial (young/fresh leaves)	October-March (October-April)	Knife
*Daucus carota* L.	*Apiaceae*	3	50% Whole aerial, 50% leaves	November-March (May-August)	17% Gardening scissor, 50% knife, 33% Hand
*Eruca vesicaria* (L.) Cav.	*Brassicaceae*	3	50% Leaves, 50% upper stem	March-May (all year)	25% Gardening scissor, 75% hand
*Melissa officinalis* L.	*Lamiaceae*	3	Upper stem	July (April-August)	Gardening scissor
*Pyrus communis* L.	*Rosaceae*	3	75% Fruit, 25% branch	June (April-June and September-October)	25% Gardening scissor, 75% hand
*Raphanus raphanistrum* L.	*Brassicaceae*	3	Whole aerial (young/fresh leaves)	November-March (November-May)	75% Knife, 25% hand
*Rubus sanctus Schreb.*	*Rosaceae*	3	75% Fruit, 25% leaves	September (July-September and January-February)	Hand
*Scolymus hispanicus* L.	*Compositae*	3	25% Whole plant, 75% whole aerial	November-February (October-April)	77% Knife, 33% hand
*Sinapis arvensis* L. subsp. *arvensis*	*Brassicaceae*	3	Whole aerial (young/fresh leaves)	November-April (May)	75% Knife, 25% hand
*Urtica* sp.	*Urticaceae*	3	67% Upper stem, 33% whole plant	April-May (March-June)	29% Gardening scissor, 71% hand
*Alkanna tinctoria* Tausch	*Boraginaceae*	2	Whole plant	August	Digging tool
*Anthriscus* sp.	*Apiaceae*	2	Whole aerial (young/fresh leaves)	November-March	77% Knife, 33% hand
*Cistus* sp.	*Cistaceae*	2	Upper stem	February-April (June)	Gardening scissor
*Ficus carica* L.	*Moraceae*	2	Fruit	July (August)	Hand
*Lactuca serriola* L.	*Compositae*	2	Whole aerial (young/fresh leaves)	October-April	63% Knife, 37% hand
*Limonium sinuatum* (L.) Mill.	*Plumbaginaceae*	2	Whole aerial (young/fresh leaves)	November-February (October-March)	75% Knife, 25% hand
*Papaver dubium* L.	*Papaveraceae*	2	Leaves	March (October-March)	Knife
*Plantago lanceolata* L.	*Plantaginaceae*	2	Whole aerial (young/fresh leaves)	February-March (October-April)	77% Knife, 33% hand
*Plantago weldenii* Rchb.	*Plantaginaceae*	2	Whole aerial (young/fresh leaves)	November-March	Knife
*Rumex obtusifolius* L.	*Polygonaceae*	2	Whole aerial (young/fresh leaves)	November-March (October-April)	75% Knife, 25% hand
*Silybum marianum* (L.) Gaertn.	*Compositae*	2	80% Flower, 20% whole plant	June-July (October-February)	80% Gardening scissor, 20% knife
*Taraxacum* sp.	*Compositae*	2	Whole aerial (young/fresh leaves)	November-March	Knife
*Acanthus spinosus* L.	*Acanthaceae*	1	Fruit	May-June	Gardening scissor
*Amaranthus retroflexus* L.	*Amaranthaceae*	1	Whole aerial (young/fresh leaves)	May-August	Hand
*Apium* sp.	*Apiaceae*	1	Whole aerial	March-July	Knife
*Ballota acetabulosa* (L.) Benth.	*Lamiaceae*	1	Upper stem	April-May	Hand
*Calendula arvensis* M.Bieb.	*Compositae*	1	Flower	April	Hand
*Cardopatium corymbosum* (L.) Pers.	*Compositae*	1	Root	May-June	Knife
*Carthamus dentatus* subsp. *ruber* (Link) Hanelt	*Compositae*	1	Whole aerial	November-March	Knife
*Centaurium pulchellum* (S*w.) Druce*	*Gentianaceae*	1	Upper stem	June	Hand
*Crataegus azarolus* L.	*Rosaceae*	1	Upper stem—including fruit and leaves	August	Gardening scissor
*Crepis zacintha* (L.) Babc.	*Compositae*	1	Seed	June-August	Hand
*Cuscuta* sp. and *Orobanche* sp.	*Convolvulaceae, Orobanchaceae*	1	Whole aerial	June-August	Hand
*Cydonia* sp.	*Rosaceae*	1	Fruit	September-October	Hand
*Cynodon dactylon* (L.) Pers.	*Poaceae*	1	Whole plant	All year	50% Gardening scissor, 50% knife
*Dittrichia viscosa* (L.) Greuter	*Compositae*	1	Leaves	July	Hand
*Echium plantagineum* L.	*Boraginaceae*	1	Whole aerial	October-March	Knife
*Erodium cicutarium* (L.) L'Her.	*Geraniaceae*	1	Whole aerial (young/fresh leaves)	November-March	Knife
*Galium aparine* L. and *Polypogon monspeliensis* (L.) Desf.	*Rubiaceae, Poaceae*	1	Whole aerial	May	Hand
*Hyoscyamus albus* L.	*Solanaceae*	1	Upper stem	July-August	Hand
*Hypericum olympicum* L.	*Hypericaceae*	1	Upper stem	May-June	Hand
*Juglans regia* L.	*Juglandaceae*	1	Fruit	October-November	Hand
*Laurus nobilis* L.	*Lauraceae*	1	Branch (with leaves)	August	Hand
*Mentha aquatica* L.	*Lamiaceae*	1	Upper stem	April-May and September-November	Hand
*Mentha spicata* L.	*Lamiaceae*	1	Upper stem	June	Hand
*Morus* sp.	*Moraceae*	1	Fruit	May	Hand
*Olea europaea* L. subsp. *oleaster* (Hoffmanns. & Link) Negodi	*Oleaceae*	1	Fruit	October-November	Hand
*Opuntia ficus-indica* (L.) Mill.	*Cactaceae*	1	Fruit	All year	Hand
*Prunus dulcis* (Mill.) D.A.Webb	*Rosaceae*	1	Fruit	September	Hand
*Quercus coccifera* L.	*Fagaceae*	1	Trunk bark	All year	Κnife
*Robinia pseudoacacia* L.	*Leguminosae*	1	Flower	May	Hand
*Rosmarinus officinalis* L.	*Lamiaceae*	1	Leaves	All year	Gardening scissor
*Rumex crispus* L.	*Polygonaceae*	1	Leaves	December-June	Κnife
*Salicornia europaea* L.	*Amaranthaceae*	1	Whole plant	March	Hand
*Sambucus nigra* L.	*Adoxaceae*	1	Flower	June	Hand
*Sinapis alba* L.	*Brassicaceae*	1	50% Whole aerial, 50% leaves	February (November-March)	Knife
*Solanum villosum* Mill.	*Solanaceae*	1	Fruit	July-August	Hand
*Solanum nigrum* L.	*Solanaceae*	1	Upper stem	November-June	Hand
*Dioscorea communis* (L.) Caddick & Wilkin	*Dioscoreaceae*	1	Upper stem	March-April	Hand
*Viola kitaibeliana* Schult.	*Violaceae*	1	Flower	April-May	Hand
*Vitex agnus-castus* L.	*Lamiaceae*	1	Upper stem	July	Hand

^a^Frequency of referrals for plants that had been harvested by the informants (*n* = 16) at least once within the last four years

^b^Percentages refer to the proportion of citations for each plant part harvested as part of the total amount of citations for each plant taxa

^c^Most frequently cited harvesting time period for each plant taxa. Parentheses indicate the less frequently cited time periods for harvesting a plant taxa

^d^Percentages refer to the proportion of citations for each harvesting equipment/tool as part of the total amount of citations for each plant taxa

**Fig. 3:**
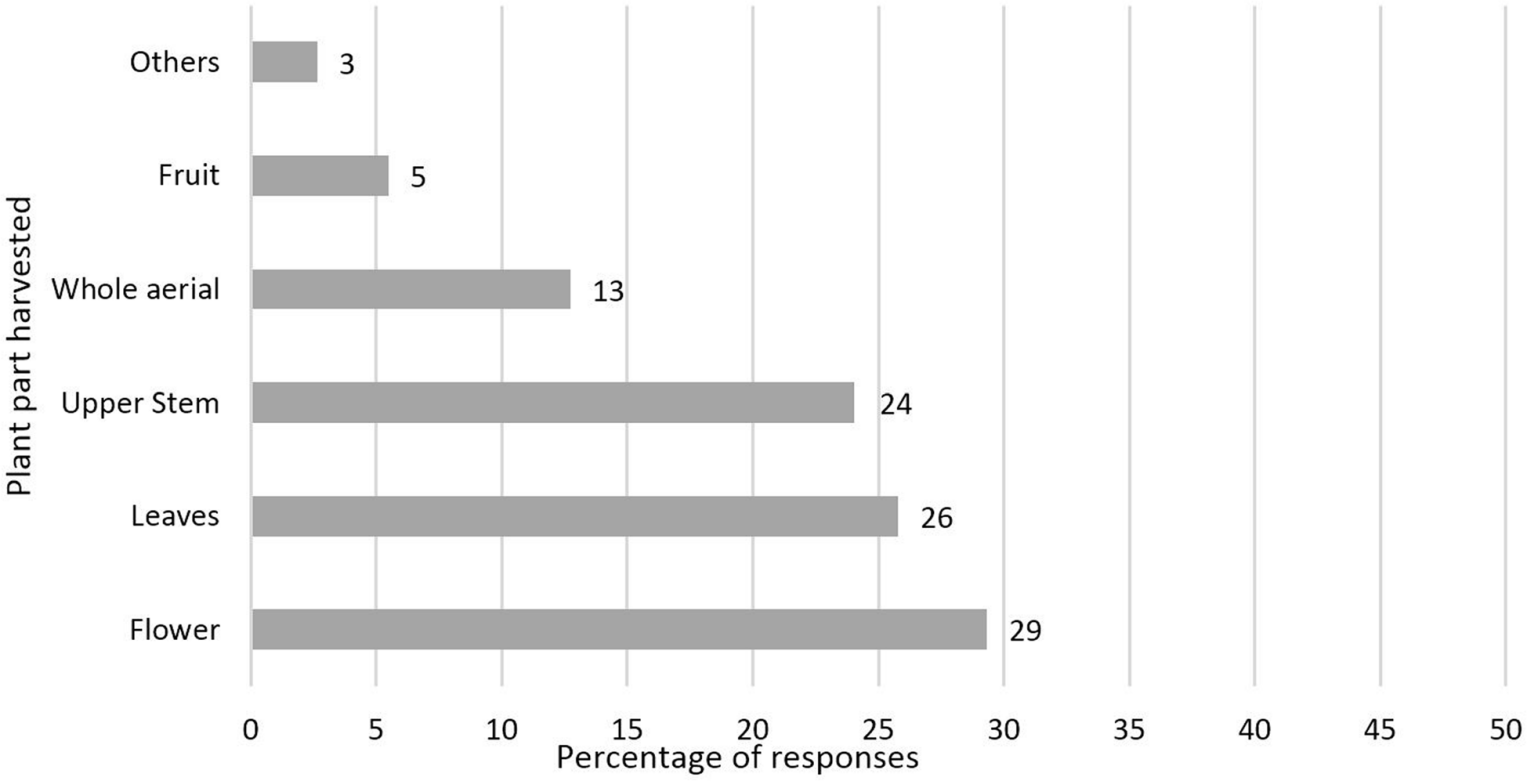
Percentage distribution of harvested plant parts (100% = 566 plant parts) (*f* ≤ 10 is summarized as “Others” including seed, branch, root, rhizome, trunk bark, and whole plant) (*n* = 16)

The most harvested plant parts were flowers, leaves (usually mentioned as young/tender leaves), and upper stem parts (including leaves and/or flowers) (Fig. 3). Plant bulbs and rhizomes were not harvested in any case while roots, trunk barks, and whole plant removals were cited only in very few cases (Table 2).

**Fig. 4:**
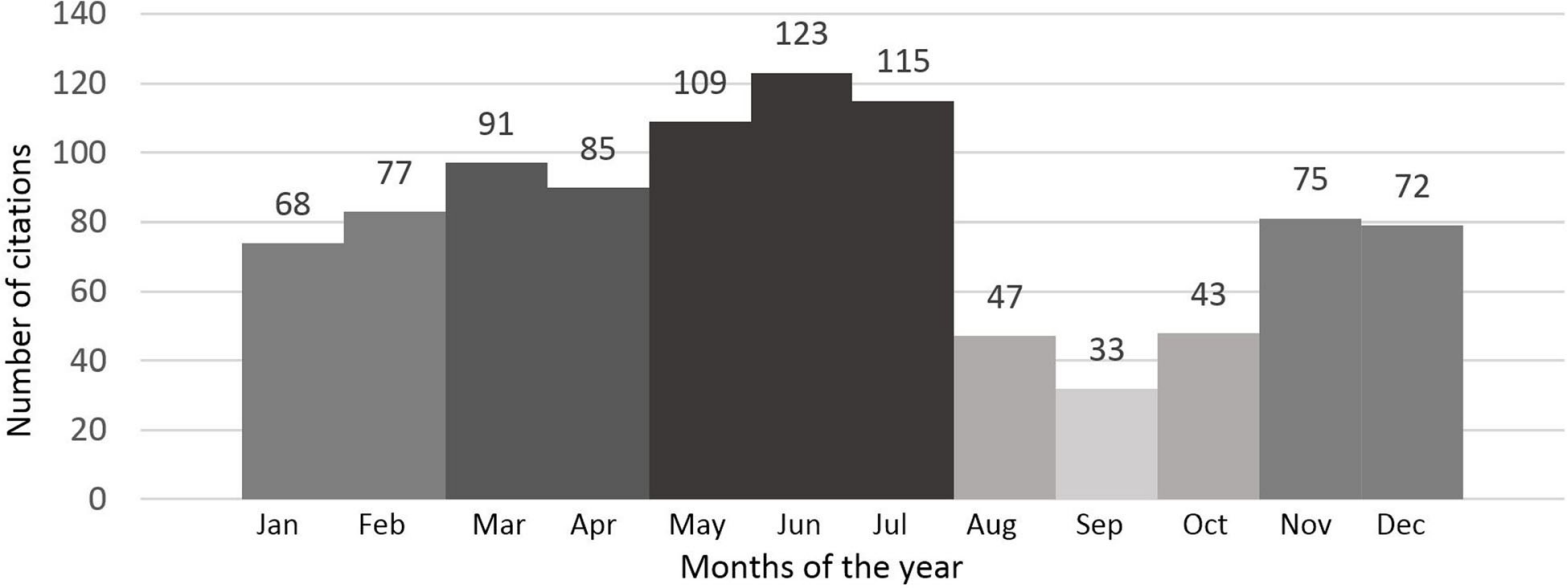
Frequency of harvesting throughout the months of the year. Numbers refer to the total of responses for each of the months (n=16). More than one response may refer to the same plant species, but a different plant part harvested, application, processing, or preparation method

Harvesting by hand was by far the most preferred method with 50% of total responses (100% = 566 harvested plant parts), followed by harvesting with a knife (26%) and gardening scissors (22%). The use of a digging tool or scythe covered the remaining 2% of the responses. The months between March and July are the busiest time of the year for WMP harvesting (Fig. 4).

Two out of the sixteen interviewees reported to dry, package, and regionally market part of their harvest to attain an additional income. The referred plant material quantities for sale varied from a few hundred grams of *T. capitata* and *O. vulgare* subsp*. hirtum* destined to a few neighbors to 70 kilograms of dried and packaged *O. vulgare* subsp*. hirtum* sold in the local market. It was observed on site that the harvested *O. vulgare* subsp. *hirtum* was at the very beginning of their blooming (one to five blooming flowers per stem). For all the remaining harvested plants and interviewed cases, the determinant of the harvested amount equated each individual’s household needs till the next possible harvest, usually one year after.

### Medicinal applications

**Fig. 5:**
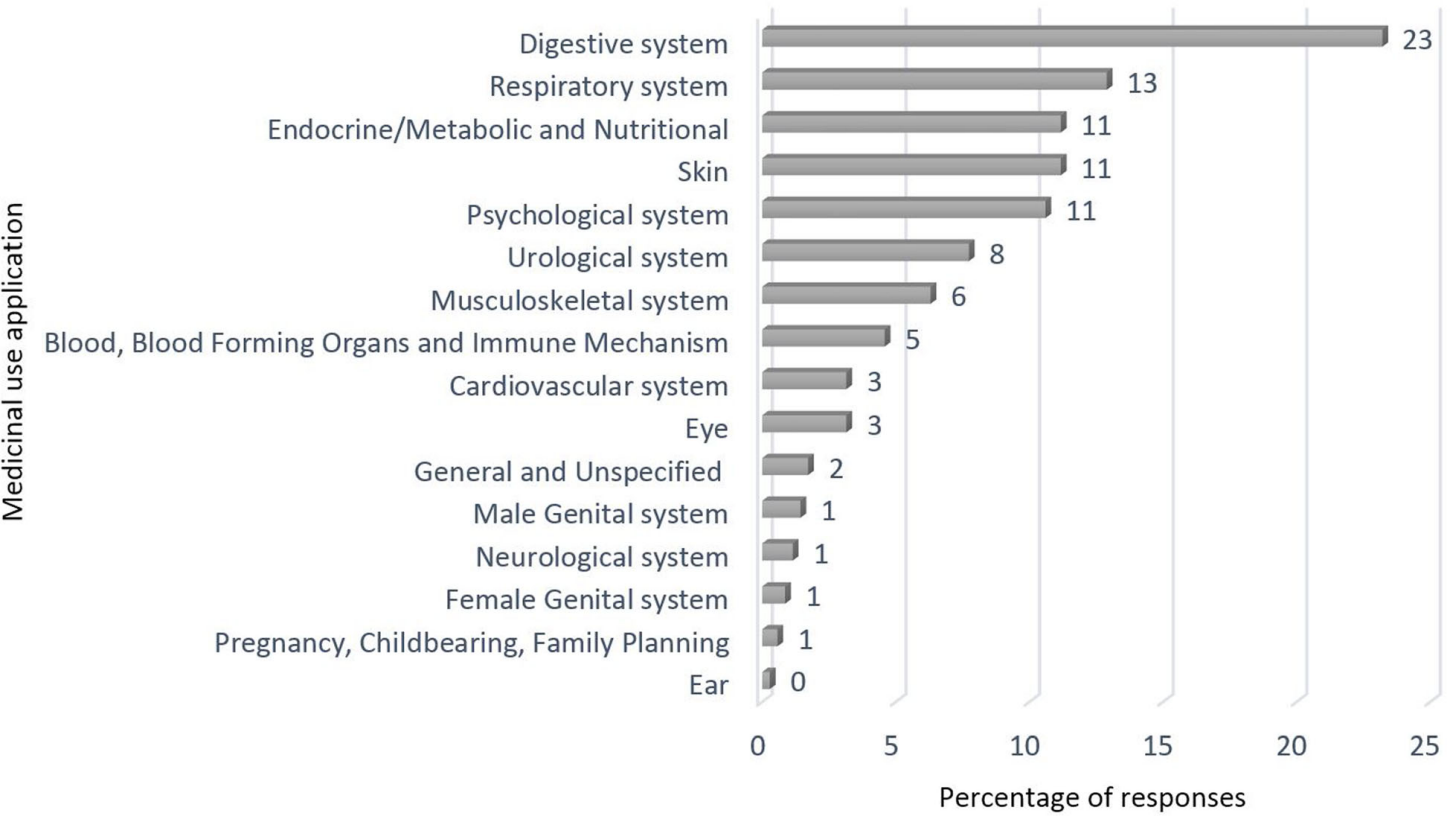
Number of medicinal use applications in the percentage of total quotes (100% = 341) (*n* = 16)

Respondents gave 341 medicinal use reports for the 81 plant taxa harvested in the ultimate 4 years. For the vast majority of plant taxa, more than one use report was assigned by harvesters (for example *M. chamomilla* presents 33 use reports). The most frequently cited applications were related to issues of the digestive (23% of all 341 use reports) and respiratory systems (13%) (Fig. 5).

From the total of 81 harvested plant taxa, 30 plant taxa were reported being used for both treatment and prevention (37%), 19 plant taxa (23%) only for treatment, and 16 plant taxa (19%) only for prevention of diseases. For the remaining 16 plant taxa (19%), respondents did not give specific medicinal properties despite assigning these plants as medicinal (Additional file 2).

Plant material from 39 plant taxa (48% of all 81 harvested plant taxa) was used fresh exclusively, 18 plant taxa (21%) were used in both fresh and processed states, and 24 plant taxa (30%) were only used after being processed. Processing was intended to preserve the harvested material in a state for future use. The most preferred method for processing was shade drying, involving more than half (54%) of the use reports with a “processed” indication (100% = 245) (Additional file 2).

Out of the 341 medicinal use reports, in 252 cases, plant material was prepared before consumption or application. Preparation methods with the highest number of mentions were infusion (44%) and boiling (34%) (100% = 252). Preparation refers to the processing of fresh or pre-processed plant material for immediate consumption or application. The end-product was most commonly consumed orally (drink or eat) (Additional file 2).

Even if people were asked to describe only the medicinal applications for each of the harvested plants, some mentioned additional uses. Species like *O. vulgare* subsp. *hirtum*, *T. capitata*, *Crithmum maritimum*, *Asparagus acutifolius* L., *Eruca vesicaria* (L.) Cav., *Cichorium intybus* L., *Portulaca oleracea* L., and several species of the genus *Taraxacum* were reported to be used mainly as food or condiment due to their pleasant taste. Almost half of the harvested plants have been recorded to be used primarily as food or flavor enhancer rather than medicine (Additional file 2).

### Sustainable harvesting practices

#### Harvest planning

Half of the respondents (50% of the sample, *n* = 16) described their WMP harvesting as a solely scheduled activity, whereas four informants (25%) characterized their harvesting as a completely unscheduled activity. The last quarter of the sample reported that wild harvesting is largely unscheduled, but not in all cases. Those harvesters scheduling their harvesting walks beforehand usually follow an annual harvest plan about when, where, and which plant species are going to be harvested. Shortly before harvesting, they visit harvesting sites and check whether plants are at the right stage of development to be harvested. They indicated that harvesting time varies from year to year and depends on weather conditions like precipitation and temperature variations. Those harvesters that do not schedule their harvest collect a WMP only in cases where it is spontaneously found in the right development stage, while taking a walk outdoors or on their way to accomplish other daily tasks.

A harvester emphasized that his/her harvest planning relies principally on information regarding plant maturity that he/she acquires throughout frequent visits at the harvesting sites. Specifically, harvesting of mature or fading flowers was mentioned oftentimes for plant taxa like *T. capitata*, *O. vulgare* subsp. *hirtum*, *Cistus* sp., *Matricaria chamomilla*, *H. perfoliatum*, *H. perforatum*, *H. triquetrifolium* Turra, *H. olympicum* L., and *Malva sylvestris*. It was said that the harvesting of these plants needs to be scheduled in advance. Harvesters mentioned that at that stage, flowers have had enough time to attract pollinators but still hold their medicinal properties and can be removed.

#### Harvesting site choice

Choosing a harvesting site that is close to home or reachable by car and easily accessible—no rough/rocky terrain—was one of the criteria people first presented when asked about what determines their choice of a harvesting site. Ten informants (62.5% of the sample, *n* = 16) stated that a potential harvesting site must be unpolluted and as clean and pristine as possible. The main sources of pollution pointed out were car emissions, road-dust, and residues of chemical pesticides and fertilizers nearby agricultural land. Other causes were garbage, stagnant and polluted waters, grazing land, high-voltage wires, and any other signs of human activity. Areas located far from those and particularly in higher altitudes were considered suitable harvesting locations as they offer uncontaminated, “clean,” and high-quality plant material.

**Fig. 6:**
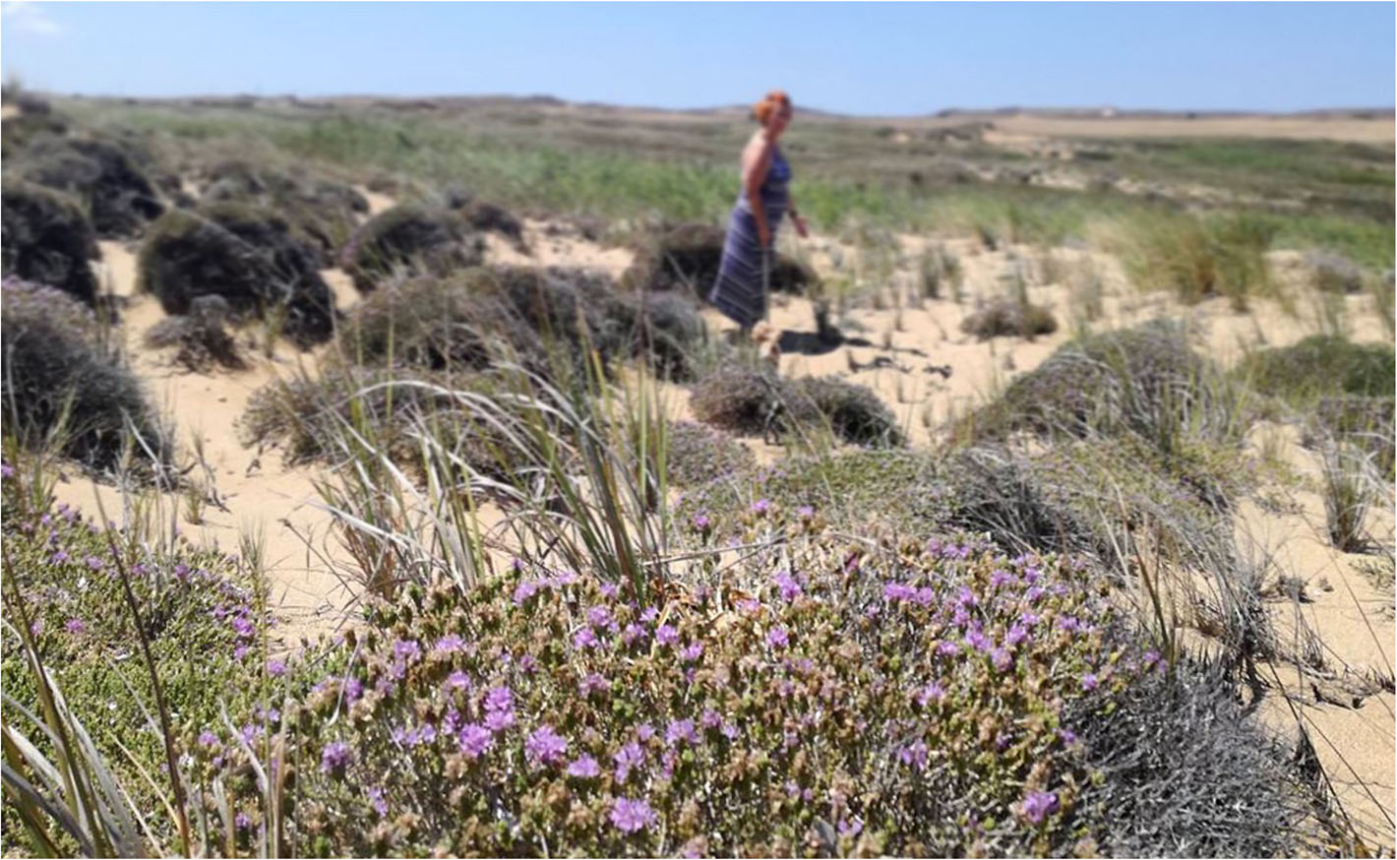
Local harvester collecting mature flowers of thyme (*T. capitata* (L.) Cav.), at clean from human activity site of northern Lemnos with high plant abundance

Three informants (18.7% of the sample, *n* = 16) said that in cases where a certain plant species is available in more than one harvesting site, they would rather choose the area with the highest abundance (Fig. 6). In such locations, some explained that plants can be prosperous and have become well established due to local conditions, therefore can tolerate harvesting much better. Others indicated that the choice of their harvesting site is a matter of experience and knowledge earned throughout time. They identified these sites as areas where they have already been harvesting multiple times in the past and therefore know which plants grow there and when. One of the interviewees said that she/he would pick the less popular site among other harvesters and supported the idea that this is a way to prevent the plant species from over-exploitation.

Other factors that came up as strong determinants while deciding on a harvesting site were the quality and maturity of the targeted plant species depending on the regional microclimate, soil conditions, and the orientation of the potential slope for harvesting. Areas of the island that receive little rainfall and present dry weather conditions (northern part) are preferred for aromatic WMPs like *O. vulgare* subsp. *hirtum* and *T. capitata* (Fig. 6). Respondents stated that under such circumstances, these plants develop higher amounts of essential oils and demonstrate higher quality organoleptic characteristics like more intense taste and stronger smell. On the contrary, areas with higher rainfall and humidity levels (Kaspakas, Aghios Demetrios, Katalako, and mainly the southern parts of the island) are preferred for wild leafy greens and asparagus. As explained, wet conditions boost the plants’ vegetative growth and promote the development of softer and tastier plant tissues like leaves.

The existence of possible additional activities around the harvesting area played a role for a few harvesters in selecting their harvesting sites. Such activities were supported by the proximity to the sea and beaches, the presence of a highly valued natural landscape, and the potential of exploring and finding new interesting medicinal or edible plant species in the area.

#### Harvesting practices inducing plant perpetuation

When informants were asked to point out examples of their harvesting practices that they consider beneficial for the perpetuation of WMP populations, all except one could mention a series of such practices. The most common statement that occurred was that the extraction of higher plant parts like ripe flowers, leaves, or the upper soft, young tissues of a plant was beneficial for plant perpetuation.

Some highlighted the importance of using gardening scissors or a knife to avoid damaging or uprooting plant individuals. One harvester indicated that using a sharp knife is essential for collecting wild leafy greens to prevent uprooting.

Harvesters pointed out that even when harvesting by hand, they are paying attention to not uproot the plant but to remove only the part that is going to be used. There were cases where harvesting was seen as a practice that boosts a plant’s regrowth, like for *O. vulgare* subsp. *hirtum*, *T. capitata*, *Matricaria chamomilla*, *H. perfoliatum*, *H. perforatum*, and *Mentha pulegium*.

Harvesting sporadically in a site, small quantities—enough to cover a household's needs for a year—and from dense plant populations were described as practices that enhance the ability of a species to establish and thrive in an area. Additionally, two harvesters reported that they adjusted their harvesting practices (location, timing, amount) according to changes in WMP availability, as to avoid depleting plant species from sites with observed small populations.

One harvester identified three harvesting techniques related to plant perpetuation, which were not mentioned at all from the other members of the sample. She/he suggested that from an individual shrub, plant material should be removed from the inner part of it and not from the edges. Similarly, on a harvesting site, plants should not be collected from the perimeter of the plant population but only from its core. She/he made clear that this is because the plant individuals at the edges are those that will contribute the most to the population expansion. She/he then added that any harvesting should occur after flower maturation, when it starts fading, since during flowering, plants have the highest essential oils’ concentrations to attract pollinators. Complementing that harvesting a plant after that stage does not affect the population’s propagation process since it increases the chances of pollen spreading.

A distinct method suggested for *M. chamomilla* and *H. perforatum* flower harvesting was the “comb-hand” technique. In this method, the harvester uses his/her palm with fingers slightly open—forming a comb-like form—so that the stem of a plant slides between them but flowers are trapped inside the harvester’s palm when pulling up. This way the needed flower heads are detached, and the remaining plant parts are left unaffected, allowing plant reproduction.

#### Harvesting practices avoided

Twelve of the harvesters emphasized the destructive effect of uprooting, when the intended plant part is not the root, and described this harvesting method as unacceptable. However, almost all of them could give an example of an island’s resident that practiced or still practices this kind of harvesting for *O. vulgare* subsp. *hirtum* or *T. capitata*.

Three harvesters reported that they do not harvest plant species that they consider rare or populations of weak plant individuals, to protect them from going extinct. In the same vein, they reported not to harvest plant species when the previous year’s harvest is still enough to cover the household needs for one more year. One harvester added on this topic that she/he prefers cultivation over wild-gathering of oregano since this and other wild plants should stay in their natural habitats and provide joy to other people passing by, as well as food to other forms of life like insects and grazing animals.

Lastly, a general higher preference towards paper or textile (cotton) bags and straw baskets was noticed. Harvesters use only or mostly these materials as they are environmentally friendly and help their harvested material stay in good condition for longer periods of time. The use of a straw basket to transport the harvested plant material was pointed out to promote plant pollination, since when freshly harvested material—flowers—is placed in a straw basket, it can easier spread its pollen and increase the chances of fertilizing other plants or leaving behind already fertilized seed during the harvesting process.

### Wild medicinal plant availability

#### Perceived availability changes

Thirteen harvesters reported a decreased availability for certain plant species in general or for specific harvesting sites. The reasons for decreased availability included increased human population density, uprooting and overharvesting, increasing use popularity, weather and climate, farming practices, and changes in land use. Only two of the sample members did not recognize any change in the availability of WMPs on the island, while another two talked about a fluctuating trend of plant populations throughout the past years due to changes in weather conditions.

The high human population density was reported to lead to over-extraction and lower medicinal plant abundance especially in the area between Myrina—capital city of the island—and Agios Demetrios. The large number of inhabitants living in this part of the island is becoming more and more familiar with the harvesting of common medicinal plants like *O. vulgare* subsp*. hirtum*, *T. capitata*, *H. perfoliatum*, and *H. perforatum* adding pressure to their populations.

Uprooting and overharvesting were identified as the most common reasons for the downturn of *O. vulgare* subsp. *hirtum* populations. While this plant subspecies was plentiful and easy to find in the past, nowadays it has become less abundant and more difficult to find. Its growing reputation as a spice and flavor enhancer on a local level during the last decade led to an increasing demand for it. Thus, more and more inhabitants started collecting it for home consumption or small-scale commercialization.

Other medicinal plant taxa reported to be threatened from overharvesting were *Crithmum maritimum*, *Capparis spinosa* L., *Salvia spp*., and *A. acutifolius*, for the consumption of which there was an increasing popularity as edibles over the last decade, which resulted in an enlarged extraction from the wild. Yet, seven harvesters reported that the unusually dry weather conditions of the last few years are equally responsible for this decline and for the general decrease of wild plant taxa populations.

Many of the informants criticized the farming practices applied on the island. The use of chemical pesticides in agriculture was also pointed out as a major polluting factor and contributor towards less availability of WMPs. Notably, for *Matricaria chamomilla*, *Malva sylvestris*, poppy species (*Papaver dubium* L., *Papaver rhoeas* L.) wild leafy greens like *Sinapis arvensis* subsp. *arvensis*, *Sinapis alba* L., and *Raphanus raphanistrum* L., as these are commonly harvested in flatlands, within or between cultivated fields.

Livestock farmers were blamed for the frequently practiced pastureland burning specifically in areas where *Sarcopoterium spinosum* (L.) Spach is dominating, in order to boost the pastureland’s grass development in the years to follow. These areas usually host *T. capitata*, the populations of which are also burned in this process. Since this species presents slow-growth rates, its population cannot recover under the pressure of heavy grazing and other highly competitive and fast-growing plant species (e.g., *S. spinosum*).

Changes in land use such as new infrastructure, farming intensification, agricultural land abandonment, and demolition of stone-hedges between fields were mentioned as additional important causes for the decrease of WMP populations in Lemnos.

#### Actions undertaken

Half of the harvesters (eight) showed their discontent with harvesters carrying out unsustainable harvesting practices and reported that in such incidents, they advised them about the consequences of their actions. A few added that when they took note of an affected population, they immediately changed harvesting sites in order to lessen the harvesting pressure at the site concerned. For the rare species of crocus (*Crocus* spp.) and orchids (*Ophrys* spp.), a respondent said that he/she never harvests them nor communicates their existence on the island to others in order to protect them from going extinct.

Two of the respondents mentioned that they cultivated a WMP species. The first case was *O. vulgare* subsp*. hirtum* for which a harvester decided upon quitting wild harvesting and only cultivating it for his/her household and market needs. A second informant reported that he/she unsuccessfully tried to cultivate striped *Crepis zacintha* (L.) Babc. seeds to deal with its reduced availability and thus the difficulty to obtain from the wild.

## Discussion

### Wild medicinal plants known, harvested, and used

In this study, the volume and depth of WMP harvesting knowledge appeared in great variance among members of the sample, meaning that none of these individuals hold the entire body of knowledge. When respondents were asked to point out knowledgeable harvesters at the end of each interview, most of them could not indicate any person on the island. This suggests that there are few knowledgeable harvesters but also little communication about WMP harvesting on the island. Although few were known to be knowledgeable, those harvesters interviewed know, harvest, and utilize a wide range of WMP taxa. At the same time, an increasing number of harvesters was described to know and harvest a few popular WMP taxa like *O. vulgare* subsp. *hirtum*, *Crithmum maritimum*, *Capparis spinosa*, and *A. acutifolius*, indicating a parallel presence of some knowledgeable harvesters with many less knowledgeable harvesters targeting certain common plant taxa only.

Our respondents also expressed their disappointment regarding the gradual loss of local knowledge regarding WMP harvesting on the island. This phenomenon was observed and widely discussed in other studies around the Mediterranean [36, 37], for example, in Albania [38], Turkey [39, 40], Spain [41], Italy [42], and Cyprus [37]. Multiple drivers were found to cause this knowledge decline, including the emerged urban lifestyles [36, 39, 42], the poverty stigma of wild plant gathering [37, 43], land use, and economic changes [5, 44].

This study of WMPs is the first that followed ethnobotanical methodology exclusively on Lemnos island, collecting information not only on the botanical level but also documenting harvesting practices, processing techniques, preparation methods, and medicinal applications for treatment or prevention of diseases and health issues. The island of Lemnos was previously included in only one ethnobotanical study, conducted by Axiotis et al. [45], that evaluated the status of traditional medicinal plant uses in the Northeastern Aegean region. From a total of 69 WMP species of Lemnos presented in their study, 34 are also occurring in this work as known by our respondents, while the rest were not cited by any of them (Table 1).

The plant families of *Compositae*, *Lamiaceae*, *Apiaceae*, and *Rosaceae* are the most represented in the list resulting from free-listing. This is not surprising, as several ethnobotanical studies around the Mediterranean [36, 46–48] and neighboring regions of Turkey [49–52] have shown that the taxonomic families with the highest occurrence of utilized medicinal plant taxa are *Compositae*, *Lamiaceae*, and *Rosaceae*. When comparing with the overall flora existing on Lemnos, all aforementioned plant families except *Rosaceae* reside within the seven most represented plant families of the island [17, 18]. As expected, some of the most popular WMP taxa in Lemnos, like *O. vulgare* subsp. *hirtum*, *C. intybus*, *Matricaria chamomilla*, *Malva sylvestris*, *Sambucus nigra* L., and *Mentha pulegium* are also present and utilized in other traditional pharmacopeias around the Mediterranean [36, 46, 47, 51, 53, 54].

### Medicinal applications

Medicinal applications for disease treatment or prevention covered the whole range of pathological domains, except the one of social problems [55]. After comparing all our findings with other studies in the Mediterranean basin we did not recognize any WMP species being specific to Lemnos. The same or very similar medicinal properties of some plants presented in the current study have been reported in ethnopharmacological studies around the Mediterranean. For example, the use of aerial parts of *F. vulgare* for health issues related to the digestive system is reported by González-Tejero et al. [36] (Algeria, Cyprus, Italy) and Sargın et al. [56] (Turkey). Roots of *C. dactylon* are used for the preparation of remedies to heal issues of the urinary system in Tunisia as well [57]. The flowering aerial parts of *O. vulgare* are traditionally consumed in Albania as a tea to treat respiratory ailments too [58]. Multiple and similar medicinal applications of *H. perfoliatum* and *H. perforatum* have been reported in another study from Greece [59].

Ethnobotanical studies conducted in neighboring regions present a plethora of similarities in terms of plant uses, plant parts utilized, processing, and application methods. The edibility of *A. acutifolius* young shoots is reported in regions of western Turkey [40, 51, 52], while its medicinal effect against kidney-related diseases is mentioned by local people of the Marmaris district [49]. The skin wart and toothache therapeutic properties of *F. carica* latex have been presented by locals of the Alaşehir region [56], the Marmara island [51], and Turgutlu district [50]. The effects of infused *Melissa officinalis* L. flowering stems as sedative and treatment against memory disturbances, coughing, and abdominal pain, that have also been reported by Axiotis et al. [45] (Greece), are cited by people living at the neighboring Edremit Gulf [60] and Kapıdağ Peninsula [61]. Same or very similar processing methods (olive oil lotion) and applications (healing of skin wounds, hemorrhoids, stomach ailments, and ulcer) of *H. perfoliatum* and *H. perforatum* are described in the works of Sargin et al. [56], Bulut [51], Ugulu [62], Uysal et al. [61], and Polat and Satıl [60]. *J. regia* leaves are applied externally for dental care purposes by inhabitants of the Marmara island [51], nearly coinciding with the teeth whitening properties of the fruit peel cited by our informants. *C. zacintha* was presented in only one nearby study [39], however attributing different preparations and medicinal uses (cut, boiled, and drunk to treat hemorrhoids versus eaten raw to treat skin warts in our study). Lastly, several plant species cited by our respondents as edibles or spices present the same usage in some of the aforementioned study areas. These include the pickled flower buds of *Capparis* spinosa [63], young stems of *Crithmum maritimum* [52, 53], *Cichorium intybus* [51, 52, 63], *Erodium cicutarium* (L.) L’Her [53]., fresh or boiled *P. oleracea* stems [40, 52, 61], *Scolymus hispanicus* L. [52, 61], *Limonium sinuatum* (L.) Mill [53]., *O. vulgare* subsp*. hirtum* (spice) [53], and *R. officinalis* (spice) [40, 52, 53].

Despite the fact that 25 plant species cited as harvested and utilized in our study are occurring in the study of Axiotis et al. [45], similarities in terms of medicinal uses are not proportionally as many. From 204 medicinal uses assigned to these 25 plant species in our work, only 19 coincide with the medicinal uses presented by Axiotis et al. [45] (Additional file 2). It is not clear though whether these medicinal uses were reported by local people of Lemnos, as the results of their study rely on information collected on a sample of 200 members inhabiting nine islands of the Northeastern Aegean region where diversity of WMP uses seems very high. Research investigating more in detail the WMP uses on each of these islands could yield promising and comparable results.

Plants with reported antioxidant content by our informants were also assigned as medicinal due to the justified ability of antioxidants to reduce oxidative stress in cells and therefore their suitability in treating many human diseases including inflammatory, cardiovascular diseases, and cancer. Additionally, natural antioxidants have also been suggested for use in preventative medicine [64].

Plants cited by at least two informants as having specific medicinal use reports were examined for their validity as therapeutic and/or preventative agents in pharmacological literature. All were found to hold pharmacological or medicinal properties, and many of which coincide or approximate the medicinal properties reported by our informants. Both *T. capitata* and *O. vulgare* have been reported to have antibacterial, antioxidant [65, 66], and antiviral [66–68] properties suggesting a potential repressive activity against bacterial and viral human body infections. Some of our informants assigned the exact same properties to these plants or suggested their use against diseases or health issues of bacterial or viral etiology (for example, common cold [69], urinary tract infection [70], diarrhea [71]). Similarly, the cited antidepressant, wound healing, and anti-inflammatory properties of Saint John’s Worts (*H. perfoliatum, H. perforatum, H. triquetrifolium*) have been justified in literature [72–76]. Equally, for the antioxidant activity of *Crithmum maritimum* [77], the kidney protective properties of *Taraxacum* and *Cichorium* species [78, 79], the nutritional value of *P. oleracea* [80], the nephroprotective properties of *S. oleraceus* [81], and the galactagogue action of *F. vulgare* [82] (Additional file 2).

### Sustainable wild medicinal plant harvesting practices

All knowledgeable harvesters interviewed in this study described several practices that revealed a developed local knowledge of the island’s WMP stock and supported the ecological sustainability of harvesting. These practices include the introduction of plant species in home-gardens, harvest sporadically at a site, extract small quantities from each site and each plant individual, avoid uprooting, avoid harvesting from weak plant populations, prefer harvesting from dense plant populations, remove upper stem parts and early-fading flowers, harvest from the core of a shrub or plant community, and use straw baskets to transport the harvested material. Indeed, the first eight of these parameters have been described in the literature as limiting the impact of harvesting on wild plant populations and meet the conditions under which an ecologically sustainable harvest can be achieved [3, 83–85].

Switching harvesting locations, timing, or harvested amount as a response to availability changes is an example of adaptive management resulting from transformative ecological cycles. Even if they did not explicitly refer to it in such terms, a few harvesters reported that they adjust their harvesting (location, timing, amount) according to current observations on WMP availability, as to avoid exhausting a plant species population. This means that observation and adaptation play a role in their harvest planning. Both have been described as essential elements of local ecological knowledge [84] for maintaining wild plants and other economically and culturally important resources and landscapes [83].

Regardless if harvesting was a scheduled or spontaneous activity, the harvesters paid attention to collecting plants at the appropriate developmental stage and preferably from sites with certain climatic and soil conditions that support the required high-quality characteristics. Especially *O. vulgare* subsp. *hirtum*, *T. capitata*, *Malva sylvestris*, *Cistus* sp., and *Matricaria chamomilla* were cited as plant taxa that need to be collected during the phase of flower maturity or flower fading. This condition decreases the ecological impact of plant removal as it allows longer periods for pollination [9, 83]. Similarly, the removal of mature plants, that have already set seeds, has been described in other studies as a condition under which an ecologically sustainable wild plant harvest can be achieved [83, 86, 87].

Harvesting sites with high WMP abundance were preferred by a proportion of interviewees. Large plant populations targeted by knowledgeable harvesters of Lemnos can better tolerate harvesting, as they manage to easier recover—especially plant species with a fast growth rate—suggesting a harvest favoring ecological sustainability [83].

Less popular sites and undisturbed areas were also preferred by several of our respondents. Both practices were found to enhance the sustainability of harvesting; harvesting sites with high popularity, characterized by an increased frequency, and intensity of harvesting were found to have raised likelihoods of overexploitation and population decline [9]; and wild plants harvested from disturbed areas were suggested to more likely encounter ecological sustainability issues due to a multiple disturbances effect [83]. So, although Lemnian harvesters avoid popular and disturbed places especially in order to get higher quality plant material, they unintentionally also reduce the pressure on plant species inhabiting these areas, hence increasing the potential towards an ecologically sustainable harvest.

Additionally, interviewees acknowledged several sources of anthropogenic destruction that negatively influence their harvesting flexibility and pose threats to wild plant stocks. These include industrial pollution and livestock residues, which are recognized risks for biodiversity [85] and have the potential to confer larger impacts than wild harvesting itself [83].

However, the concentration of many gatherers in narrowly defined harvesting sites recognized as clean and unpolluted may have implications for the ecosystem. This is based on the idea that the higher the number of people collecting a plant species in a confined area, the lesser possibilities for the plant populations to survive there, with the chances getting even lesser when unsustainable harvesting is practiced [9]. Such a remark was made by some members of our sample that noted a lower WMP abundance between Myrina and Agios Demetrios, blaming over-extraction of plants due to high human population density. So, even if harvesting is practiced on an individual level under ecologically sustainable terms, it is not the same on the community level for that area.

The introduction of WMPs in home gardens as a reaction to overexploited or disturbed plant populations and habitats also favors the ecological sustainability of WMP. Schippmann et al. [88] described this practice as a priority conservation option for threatened or vulnerable wild plant species, which might be the case for *Salvia* spp. and *C. zacintha*, according to our respondents. The pressure on a wild plant species is expected to be relieved if the locals’ demand can be met from cultivated sources, allowing the recovery of the plant population in the wild. Nevertheless, for wild plant species that do not face availability issues, like most of the listed plants in this study, sustainable harvesting is the best option to ensure local benefits, maintenance of populations, and ecosystem’s balance [88].

In terms of plant parts extracted, wild plant harvesting in Lemnos tends to be ecologically sustainable as the removal of roots, wood, bark, bulbs, apical meristems, phloem saps, and whole plants [83] was avoided almost entirely. Among the seventeen most harvested medicinal plant taxa only in two cases, the plant part harvested combined with the plant’s life form suggests a medium susceptibility to overcollection (Additional file 3). The first case refers to *M. chamomilla*, whose flower collection may affect its ability to perpetuate, as it is an annual plant species with its flower being the only reproductive structure [83, 88, 89]. So, the argument of some harvesters that the “comb-hand” technique benefits the plant’s regeneration compared to non-harvesting cannot be evaluated as valid in these terms. Similarly, the removal of the whole aerial part of the annual/biennial *S. oleraceus* raises the species’ vulnerability to overcollection and thus lowers the chances of an ecologically sustainable harvest.

The remaining two practices of harvesting from the core of a shrub or plant community and using straw baskets to transport the harvested material were not found in literature before. The certainty and detailed explanation of harvesters about the contribution of these practices to the sustainability of harvesting revealed their conviction about the positive effects of these practices. Several other management practices to promote plant regeneration like clearing weed, and/or light competitors to increase survival and growth of WMP species, planting, or nurturing seedlings and seeds in the wild to expand populations [84, 90] were cited by none of the informants.

### Perceived wild medicinal plant availability changes

According to harvesters interviewed, *O. vulgare* subsp. *hirtum*, *Crithmum maritimum*, *Capparis spinosa*, and *A. acutifolius* already face survival problems due to an increased popularity for their consumption as edibles and consequently a rise in the number of harvesters targeting only these taxa. So, even though the knowledgeable harvesters do not currently pose a threat while harvesting these plant taxa, the growing popularity among locals may affect, or may already have affected, its survival rates. This comes to agree with Posthouwer’s et al. [85] recommendation that the harvest of species with high use popularity may encounter sustainability problems. Similarly, Ticktin and Johns [91] indicate that wild plant populations managed by less knowledgeable harvesters may decline even under low harvest levels.

Respondents also reported a decreased availability for *M. chamomilla*, *T. capitata*, and wild leafy greens that were victims to other types of management or disturbance, in addition to harvesting. The extensive use of chemicals in agriculture was said to be responsible for less *M. chamomilla* and wild leafy greens including *S. oleraceus*. *T. capitata* was cited to be under pressure from intentional pastureland fires. These fires, combined with the species slow-growth and the highly competitive and fast-growing antagonistic plant species like *S. spinosum*, increase its vulnerability and resilience to harvesting. In this context of multiple disturbances, it can be difficult to draw clear conclusions about the factors influencing population changes [88]. For such cases prohibiting or regulating harvesting by assuming that it is the cause of population decline could have adverse consequences for the local community and would be inefficient in terms of conservation.

## Conclusion

Knowledgeable harvesters of Lemnos were found to gather and utilize a rich diversity of plant taxa. Almost all of them think about, take measures, and care about sustainable harvesting. Their knowledge on WMP harvesting embodies a considerable number of observations and practices strongly relating to an ecologically sustainable harvest. Harvesting practices showed a developed ecological understanding of the island’s WMP stock.

Our local knowledge-based assessment of the ecological sustainability of harvesting pointed out certain plant taxa and areas of the island that are under pressure from overharvesting, unusual climatic conditions, and agricultural practices like chemical inputs and intentional pastureland burning. The increasing number of less knowledgeable harvesters targeting common plant taxa and applying destructive harvesting practices, such as uprooting, may cause serious implications to plant population growth and is a major issue to be addressed.

Countermeasures against deteriorating sustainability of WMP harvesting could be based on awareness-raising and education of the local community about sustainable harvesting practices that shelter WMP and about the outside factors impeding sustainability.

This could be accomplished by introducing workshops through schools or local associations and planning sustainable harvesting seminars and field trips with experts. In addition, the few already existing eco-tourism facilities of Lemnos may include a new theme for visitors, introducing them to the rich wild medicinal flora of the island. The trekking-friendly terrain and an already existing network of trails all around the island can easily host such activities, hence maintaining the cultural character of the landscape, increasing the value of natural ecosystems, while simultaneously achieving ecological sensitization and financial benefits for the local community.

Considering that local knowledge is an integral part of cultural heritage and harvesting practices embedded in it form a link between harvesters and their ancestors, history, land, and environmental philosophy, it is important to support and encourage projects that can contribute to protecting and preserving it [92]. This study shows that ecological sustainability and WMP harvesting are not mutually exclusive, can coexist, and contribute significantly to sustaining an appropriate balance for both while keeping the culture alive.

Our approach thus confirmed that local knowledge should be taken into account for assessing the sustainability of WMP harvesting [83]. Future research, management strategies, or conservation plans seeking to protect and maintain the island’s medicinal plant populations and biodiversity can take this information as the starting point.

## Supplementary information

**Additional file 1.** Interview guide

**Additional file 2. **List of wild medicinal plants and corresponding medicinal applications reported from Lemnos knowledgeable harvesters (*n*=16).

**Additional file 3. **Susceptibility assessment of the most frequently collected wild medicinal plants taxa (f>3) to overcollection (*n*=16).

## Data Availability

All data generated during this study—except the transcripts of semi-structured interviews—are included in this published article and its additional files.
