# Supplementary material for: Local knowledge about sustainable harvesting and availability of wild medicinal plant species in Lemnos island, Greece
Source: J Ethnobiol Ethnomed. 2020 Jun 19;16:36. doi: 10.1186/s13002-020-00390-4 (PMC7304145; doi:10.1186/s13002-020-00390-4)
Supplement: Supplementary file 1 — Additional file 1. Interview guide [file 13002_2020_390_MOESM1_ESM.docx]

Additional file 1: Interview guide

1. Informant details
   1. Interviewee code:
   2. Gender: F □ M □
   3. Year of birth:
   4. Highest education level accomplished:
2. Local knowledge on ecologically sustainable wild harvesting of medicinal plants
   1. Existence and development of local knowledge
      1. Can you give me a short overview about where did this experience come from in your case?
   - When did you start wild medicinal plant harvesting?
   - How did you learn about how to recognize, harvest and use them?
   1. Harvesting sites
      1. Can you describe me actions you always follow before you go for a common wild medicinal plant harvest?
   - Is your harvest something planned or something spontaneous?
   - Where do you go for harvesting?
     1. Can you think and tell me what makes you choose a specific site for wild medicinal plant harvesting?
   - What makes you decide on a harvesting site instead of another when the targeted specie is present in both?
   1. Harvesting practices
      1. Perhaps you could tell me a little more about your harvesting practices and techniques now. Is there something you do while harvesting to ensure that you will find wild medicinal plants to harvest in the next harvesting season?
   - Do you pay attention on how you remove the plants or specific plant parts?
     e.g. bulb, stem, branches, flowers, fruits, leaves, roots, seeds?
   - Is there something you do to promote the growth of plants?
   - Is there something you do to sustain or expand the population of plants?
     1. I guess not everybody practices wild harvesting the same way like you..
   - Do you avoid any particular harvesting methods that others follow? Can you describe some of those?
   - What makes you avoid these methods?
   - Can you think of harvesting practices that could threaten the wild medicinal plants of Lemnos (ability to reproduce or regrow)?
   - Do you recognize something that makes your harvesting practices more friendly to nature? Can you be more detailed on it?
     1. Can you tell me what determines the amount of the material you will harvest from a specific site?
   - How do you decide that your harvested material of a species is enough?
   - When do you stop harvesting from a specific site?
     1. Could you think of very special and distinct harvesting technique that you practice for a specific plant?
   - Why is this technique necessary?
   1. Environmental awareness/ ecological consciousness
      1. How did the availability of wild growing medicinal plants change on your gathering sites and on the island over the last few decades?
   - Are there wild medicinal plants that were easy to find on the island in the past but not anymore?
   - Did you do something about this issue when it occurred?
   - Is there something in your mind that could explain why some plants are not easy to find anymore?
3. Successive free-list: Medicinal plants known

Can you please list all wild medicinal plants you know that grow in Lemnos?

1. Medicinal plants wild harvested and used
   1. Could you tell me which of those plants that you know did you harvest in Lemnos at least once within the last four years?

      Now that we have learned about which plants you harvest, let’s take them one by one and see how you harvested and used them the last time it happened. Follow up questions for each one of the harvested plants:
   2. Harvesting
      1. Which plant parts did you harvest?
      2. Which months of the year did you harvest it?
      3. What equipment did you use?
      4. What equipment did you use to store and transport it back to your home?
   3. Utilizing
      1. Is XY used as treatment or preventative?
         If treatment - For which illness or health abnormality is it used?
         If preventative - For which illness or health abnormality is it used?
      2. Which part of the plant is used?
      3. Is it used fresh or conserved?
      4. If conserved - How is it conserved/preserved?
      5. How is XY prepared for use?
      6. How is the preparation applied?
      7. How often do you use XY?
   4. Why do you not harvest the other listed wild medicinal plant species such as XZ, YZ or ZA?
   5. Is there anything else you would like to add?
2. Sample development
   Could you recommend other people that harvest a lot of wild growing medicinal plants on the island and know a lot about them and their uses?
