# Supplementary material for: Local knowledge about sustainable harvesting and availability of wild medicinal plant species in Lemnos island, Greece
Source: J Ethnobiol Ethnomed. 2020 Jun 19;16:36. doi: 10.1186/s13002-020-00390-4 (PMC7304145; doi:10.1186/s13002-020-00390-4)
Supplement: Supplementary file 2 — Additional file 2. List of wild medicinal plants and corresponding medicinal applications reported from Lemnos knowledgeable harvesters (n=16). [file 13002_2020_390_MOESM2_ESM.docx]

Additional file 2: List of wild medicinal plants and corresponding medicinal applications reported from Lemnos knowledgeable harvesters (n=16).

| **Scientific name** | **f** | **Plant part used** | **Mode of use** | **Processing mode** | **Preparation mode** | **Application mode** | **Medicinal use for treatment** | **Medicinal use as preventative** | **Mentioned in literature** |
| --- | --- | --- | --- | --- | --- | --- | --- | --- | --- |
| *Thymbra capitata* (L*.)* Cav. ^a^ ^b^ | 15 | Flowers and/or upper stem ^a^ | Processed | Shade dried | Aqueous infusion | Drink | Throat ache (4)  Common cold (4)  Relieves from issues of the respiratory system (1)  Diarrhea (1)  Stuffed nose (1)  Coughing ^a^ - Expectorant, Antitussive (1)  Flatulence (1)  Mouth infections (1)  Memory (1) | Antibacterial / bacteriostatic (4)  Sedative / calming agent (2)  Common cold (1)  Immune system enhancement (1)  Viricidal (1)  Boosts memory (1) | Antibacterial, antioxidant [1]  Antimicrobial [2]  Antiviral (slight) [3] |
|  |  |  |  |  | Aqueous infusion | Skin application | Skin wounds (1)  Antifungal (1)  Urinary tract infection (1) |  |  |
|  |  |  |  |  | - | Eat | Diarrhea (1) |  |  |
|  |  |  |  | Tincture (in pure pharmaceutical alcohol) | - | Skin application | Joints complains (1) |  |  |
|  |  |  |  | Tincture (in grape distillate) | - | Skin application | Common cold (1) |  |  |
|  |  |  |  | Tincture (In grape distillate with the addition of honey to become like syrup) | - | Drink | Relieves from issues of the respiratory system (1)  Diarrhea (1) |  |  |
| *Origanum vulgare* L. subsp. *hirtum* (Link) letsw. ^a^ ^b^ | 12 | Upper stem | Fresh | - | - | Inhale | Relieves from issues of the respiratory system (1) |  | Antibacterial, antioxidant [1]  Antiviral [2, 4] |
|  |  |  |  |  |  | Skin application/rubbing | Painful joints (1) |  |  |
|  |  |  | Processed | Shade dried and crushed | Aqueous infusion | Drink | Diarrhea (3)  Irritated throat (1)  Common cold ^a^ (1)  Antiviral (1)  Constipation (1)  Germicide (1)  Fungicide (1) | Antiseptic (2)  Improves stomach robustness (1)  Immune system enhancement (1)  Common cold (1)  Antibacterial (1)  Antiviral (1)  Germicide (1)  Fungicide (1) |  |
|  |  |  |  |  | As processed | Eat |  | Improves stomach robustness (1) |  |
|  |  |  |  |  | Cooked in food | Eat |  | Improves stomach robustness (1)  Immune system enhancement (1) |  |
|  |  |  |  | Shade dried | Aqueous infusion | Gargle | Relieves from issues of the respiratory system (1) |  |  |
|  |  |  |  |  |  | Drink |  | Improves stomach robustness (1)  Immune system enhancement (1) |  |
|  |  |  |  |  | - | Eat |  | Improves stomach robustness (1)  Common cold (1) |  |
|  |  |  |  |  | Cooked in food | Eat |  | Improves stomach robustness (1)  Common cold (1) |  |
|  |  |  |  |  | Lotion: 200ml warmed up extra virgin olive oil + 1 teaspoon of crushed material | Skin application/rubbing | Fever (1) |  |  |
|  |  |  |  | Essential oil extract | Diluted in water; concentration: 1ml of essential oil in 1l of water | Drink |  | Improves stomach robustness (1)  Immune system enhancement (1) |  |
|  |  |  | Fresh or processed | Shade dried | Aqueous infusion (3-10 min) | Drink | Indigestion ^a^ (1) |  |  |
|  |  |  |  |  |  | Mouth wash | Mouth infections: aphthous stomatitis, gingivitis (1) |  |  |
|  |  |  |  | Tincture | As processed | Mouth wash | Mouth infections: aphthous stomatitis, gingivitis (1) |  |  |
| *Hypericum perfoliatum* L. and *Hypericum perforatum* L. ^a^ | 11 | Flowers ^a^ | Processed | Shade dried | Aqueous infusion | Drink | Stomachache (4)  Stress (1)  Depression (1)  Insomnia (1) | Sedative/calming agent (1) | Antimicrobial, antioxidant [5, 6]  Antidepressant [7–9] |
|  |  |  |  | Shade dried & Olive oil lotion; Soak plant material in extra virgin olive oil in shade for 1 month, then strain. No sun because olive oil is oxidized this way and the preparation quality is then | - | Skin application | Skin burns ^a^ (skin regeneration) (1)  Skin wounds ^a^ (1) |  | Wound healing, anti-inflammatory [10]  Wound healing, scar of Cesarean [11] |
|  |  |  |  | Fresh & Olive oil lotion | - | Drink | Peptic ulcer disease (1)  Stomachache (1)  Purifies the blood (1)  Wounds of the digestive system/canal ^a^ (1) |  |  |
|  |  |  |  |  |  | Skin application | Skin wounds (5)  Skin irritation (2)  Skin burns (skin regeneration) (1)  Arthritis (1)  Painful joints (1)  Sore muscles (1)  Dermatitis (1) |  |  |
|  |  |  |  |  |  | Ear drops | Otitis (1) |  |  |
|  |  | Upper stem | Processed | Shade dried | Aqueous infusion | Drink | Stomach ulcer (1) |  |  |
|  |  |  |  | Tincture | - | Skin application | External hemorrhoids (1) |  |  |
|  |  |  |  | Olive oil lotion | - | Drink | Depression (1) |  |  |
|  |  |  |  |  |  | Skin application | Skin wounds (4)  Painful joints (1)  Skin burns (1)  Callus (1)  External hemorrhoids (1)  Arthritis (1)  Common cold (1) | Arthritis (1) |  |
| *Matricaria chamomilla* L. ^a^ | 10 | Flowers ^a^ | Processed | Shade dried | Aqueous infusion | Drink | Stomachache (4) Sedative/calming agent ^a^ (3)  Throat ache (2)  Insomnia (2)  Expectorant (1)  Indigestion (1)  Common cold (1)  Diabetes (1)  Anxiety (1)  Digestive system/canal wounds (1) | Sedative/calming agent (4)  Insomnia (1)  Stomach sedative ^a^ (1) | Antifungal [12]  Antioxidant [13]  Antibacterial  [2] |
|  |  |  |  |  |  | Gargle | Pharyngitis (1)  Tonsillitis (1) |  |  |
|  |  |  |  |  |  | Eye wash | Ophthalmological infections (6)  Allergic eye irritation (1)  Eye irritation (1)  Eye gum for infants (1)  Eye pain (1)  Conjunctivitis (1) |  |  |
|  |  |  |  |  |  | Skin application | Irritated skin (2)  Antiseptic ^a^ for skin wounds (2)  Acne sedative (1) |  |  |
|  |  |  |  |  |  | Wash (vaginal) | Antifungal for female genitals (1) |  |  |
|  |  |  |  |  | Aqueous infusion (Inside a tall, long bucket you add a handful of flowers and then water in boiling temperature.) | Steaming (the woman stands above the bucket covered with a bathrobe, without undergarment and with her legs open so that the steam can reach her genitalia) |  | Antifungal for gynecological/vulvar system (1) |  |
|  |  |  |  |  | Lotion (half of a handful of dried material + half glass of extra virgin olive oil. Warm it up for 10-15 min. Let it sit for 15min and use | Skin application | Sedative/calming agent (1)  Bone pain for adults (1)  Common cold (1)  Muscle back-pain (1) |  |  |
|  |  |  |  |  | Lotion (a handful of dried material + a full glass of extra virgin olive oil. Warm it up for 10-15 min. Let it sit overnight. Strain and store in glass bottle. | Skin application |  | Sedative/calming agent (1) |  |
|  |  |  |  | Shade dried and processed into a lotion (dried material sits inside extra virgin olive oil for a month in the Shade) | - | Skin application | Irritated skin (1)  Calming agent for babies (1) |  |  |
| *Mentha pulegium* L. ^a^ | 10 | Upper stem | Processed | Shade dried | Aqueous infusion | Drink | Coughing (4)  Stomachache ^a^ (3)  Common cold (2)  Indigestion (1)  Abortive (1)  Painful joints (1)  Expectorant (1)  Drunkenness (1)  Stuffed nose (1)  Flatulence (1) | Stimulant for men (1)  Rich in calcium, iron, magnesium (1)  Sedative/calming agent (1)  Common cold (1)  Respiratory diseases (1) | Bacteriostatic, bactericidal [14] |
|  |  |  | Fresh or processed | Shade dried | Aqueous infusion | Drink | Coughing (1) |  |  |
|  |  |  | Fresh or processed | Shade dried | Raw | Add plant material enough to cover the inside bottom part of the shoe. Then wear the shoe. | Tired/painful feet (1) |  |  |
| *Malva sylvestris* L. ^a^ | 8 | Flowers ^a^ | Processed | Shade dried | Aqueous infusion | Drink | Throat ache (2)  Coughing (2)  Common cold (1) | Emerging common cold (2)  Respiratory diseases (1)  Sedative/calming agent (1)  Stomach and intestines sedative (1) | Antibacterial [15]  Antimicrobial (CPC combined mouthwashes) [16] |
|  |  |  | Fresh or processed | Shade dried | Aqueous infusion | Drink | Common cold (1)  Expectorant ^a^ (1)  Sedative/Calming agent (1) |  |  |
|  |  |  | Fresh | - | Aqueous infusion | Drink |  | Digestive system (1) |  |
| *Salvia spp.* | 7 | Upper stem (leaves) | Processed | Shade dried | Aqueous infusion | Drink | Hypertension (1)  Common cold (1)  Coughing (1)  Indigestion (1)  Drunkenness (1)  Fever (1) | Antioxidant (1)  Good for heart (1) | Antimicrobial,  antioxidant, anticholinesterase, improvement of cognitive  performance and mood, reducing work-related stress,  antimutagenic, anticancer, anti-inflammatory, choleretic  activities [17]  Antibacterial  [2] |
|  |  |  |  |  |  | Inhale | Common cold (1)  Coughing (1) |  |  |
|  |  | Upper stem (flowers) | Processed | Shade dried | Cook | Eat | Helps against free radicals in human body (1) |  |  |
|  |  |  | Fresh or processed | Shade dried | Aqueous infusion | Drink | Helps against free radicals in human body (1) |  |  |
|  |  | Upper stem (flowers and leaves) | Processed | Shade dried | Aqueous infusion | Drink | Common cold (2)  Coughing (1)  Flatulence (1) | Sedative/calming agent (1) |  |
|  |  | Flowers | Fresh or processed | Shade dried | Aqueous infusion | Drink | Hypertension (1) |  |  |
| *Taraxacum* spp. ^b^ (Pikroráðiko, Taraksáko, Aɣriomárulo, Raðíki Taraksáko, Pikralíða) | 7 | Whole aerial | Fresh | - | Boil | Drink the decoction and/or Eat the boiled plant material (mainly as salad) | Kidney stone disease (1) | Antioxidant (1)  Cleans toxins from human body (1)  Cleans the liver and is very good for the kidney’s function (1)  Kidney stone disease (1)  Purifies the blood (1) | Diuretic [18] |
|  |  |  | Fresh or processed | Fridge preserved | Boil | Drink the decoction and/or Eat the boiled plant material (mainly as salad) | Diabetes (1) |  |  |
|  |  | Flower | Fresh | - | Raw | Skin application | Skin warts/Myrmecia (1) |  |  |
| *Crithmum maritimum* L. ^b^ | 6 | Upper stem (with leaves) | Processed | Pickled | - | Eat |  | Rich in vitamins and nutrients (1)  Common cold (1) | Antibacterial [19]  Antioxidant [20] |
|  |  | Leave | Processed | Shade dried | Aqueous infusion | Drink |  | Antioxidant (1) |  |
|  |  | Leave | Processed | Pickled | - | Eat | Scurvy/vitamin deficiency (1) | Antioxidant (1) |  |
| *Portulaca oleracea* L. ^b^ | 6 | Upper stem (leaves) | Fresh | - | Raw | Eat |  | Rich in omega-3 fatty acids (1) | Nutritius, Antioxidant [21]  Anti-inflammatory (potential therapeutic effect against arthritis) [22] |
|  |  |  | Processed | Pickled | - | Eat |  | Rich in omega-3 fatty acids (1) |  |
|  |  |  |  | Lotion  (plant material + extra virgin olive oil inside a glass bottle. Let in Shade for 40 days) | - | Skin application | Rheumatoid arthritis (1) |  |  |
|  |  |  | Fresh or processed | Pickled | Raw or process | Eat | Osteoporosis (1)  Aphrodisiac (1)  Dementia (1) | Osteoporosis (1)  Dementia (1)  Good for digestive system (1)  Rich in omega-3 fatty acids (1)  Rich in magnesium and minerals (1)  Aphrodisiac (1) |  |
|  |  |  |  | Pickled | Raw or process |  |  |  |  |
|  |  | Upper stem (flowers and leaves) | Processed | Pickled | - | Eat | Constipation (1) |  |  |
|  |  |  | Fresh | - | Boil | Eat |  | Rich in omega-3 fatty acids (1) |  |
|  |  | Leaves | Fresh | - | Raw | Eat |  | Cleans the blood (1)  Good for digestive system (1) Rich in omega-3 fatty acids (1) |  |
| *Sonchus oleraceus (*L.) L. ^b^ | 6 | Whole aerial | Fresh | - | Boil | Drink the decoction and/or eat the boiled plant material (mainly as salad) | Kidney stone disease (1) | Kidney stone disease (1)  Purifies the blood (1) | Antidiabetic, antioxidant [23]  Antioxidant, anti-inflammatory, nephroprotective against kidney ischemia-reperfusion [24] |
| *Rosa canina* L. ^a^ | 5 | Fruit ^a^ | Fresh or processed | Shade dried | Crush, Aqueous infusion | Drink |  | Rich in vitamin C (2)  Stimulant (1) | Anti-inflammatory [25]  Antioxidative (due to vitamin C, polyphenolics content) [26] |
|  |  | Flowers | Processed | Shade dried | Crush, Maceration | Drink | Common cold (1) | Rich in vitamin C (1)  Common cold (1) |  |
|  |  |  | Fresh | - | Aqueous infusion (petals) | Skin application (Prevention: add in the infusion a few drops of extra virgin olive oil, mix and store as ice-cubes in refrigerator) | Skin wounds ^a^ (1) | Face skin moisturizing, softening and tightening (1) |  |
|  |  | Branch bark ^a^ | Fresh | - | Boil | Drink | Arthritis (1) |  | Anti-inflammatory, anti-obesity, anti-diabetic, positive effect on osteoarthritis, rheumatoid arthritis and cancer [27] |
| *Tordylium apulum* L. ^a^ ^b^ | 5 | Whole aerial ^a^ | Fresh | - | Boil | Eat |  | Purifies the blood (1)  Antioxidant (1) | Antibacterial [28]  Antioxidant [29] |
| *Asparagus acutifolius* L.  ^a^ ^b^ | 4 | Upper stem (unripe flower buds) | Fresh | - | Raw | Eat |  | Good for kidney health/diuretic (1) | Antioxidant, antiproliferative [30] |
|  |  |  |  | - | Boiled or cook | Eat | Purifies the blood (1) | Good for kidney because of bitter taste (1) |  |
|  |  |  |  |  | Boil | Drink the decoction and/or eat the boiled plant material | Cleans the kidney (1) | Cleans the kidney (1) |  |
| *Capparis spinosa* L. ^a^ ^b^ | 4 | Unripe flower buds | Processed | Pickled | - | Eat | Spermatogenetic (1) | Appetizing (1)  Tonic (1)  Goof for urinary system (1) | Liver pain, irregular urination, impotency, aphrodisiac [31] |
| *Foeniculum vulgare* Mill.  ^a^ ^b^ | 4 | Upper stem (leaves ^a^) | Fresh | - | Cook (fish, savory pies, dolma and broad beans) | Eat |  | Good for kidney (1)  Good for stomach (1) | Antioxidant, cytotoxic, anti-inflammatory, antimicrobial, bronchodilatory, estrogenic, galactagogue, oculohypotensive, antithrombotic, hepatoprotective, effects on memory and cognition, hypotensive, antimutagenic [32]  Antibacterial  [2] |
|  |  | Upper stem (flowers and leaves) | Fresh | - | Cook | Eat | Indigestion (1) |  |  |
|  |  | Leaves and seeds | Processed | Shade dried | Aqueous infusion | Drink | Dissolves body fat (1) | Increases breast milk production (1)  Good for stomach (1) |  |
| *Hypericum triquetrifolium* Turra | 4 | Upper stem (flowers and leaves) | Processed | Lotion (Transparent glass bottle full of plant material + extra virgin olive oil. Let in sun for min 40 days. Stir 1-2 times/day) | - | Skin application | Skin wounds (2)  Painful joints (1)  External hemorrhoids (1)  Throat ache (external application) (1) |  | Antioxidant, antibacterial, antifungal [33, 34] |
|  |  | Flowers | Processed | Lotion | - | Skin application | Skin wounds (1)  Painful joints (1)  Psoriasis (1) |  |  |
| *Cichorium intybus* L. and *Cichorium pumilum* Jacq. ^b^ | 3 | Whole aerial | Fresh | - | Boil | Eat | Kidney stone disease (1)  Diabetes (1) | Kidney stone disease (1)  Purifies the blood (1) | Urinary tract, kidney diseases [35]  Hypoglycemic, hepatoprotective, antioxidant and immunomodulatory [36] |
|  |  |  |  |  |  | Drink | Kidney stone disease (1) | Kidney stone disease (1) |  |
| *Cichorium* spp. and *Taraxacum* spp. *(*Raðíkia*)* ^b^ | 3 | Whole aerial | Fresh | - | Boil | Drink the decoction and eat the boiled plant material as salad | Diabetes (1) | Source of vitamin A (1)  Good for stomach health (1) | Cichorium: Urinary tract, kidney diseases [35]  Hypoglycemic, hepatoprotective, antioxidant and immunomodulatory [36]  Taraxacum: Diuretic [18] |
|  |  |  |  |  |  | Eat |  | Good for urinary system (1) |  |
| *Daucus carota* L. ^b^ | 3 | Whole aerial | Fresh | - | Boil (salad) or cook (savory pies) | Eat |  | Good for the eyes (1)  Good for the blood (keeps the hematocrit in normal levels) (1) | Antioxidant, anticancer [37]  Antibacterial  [2] |
| *Eruca vesicaria* (L.) Cav. ^b^ | 3 | Leaves | Fresh | - | Raw (salad) | Eat | Aphrodisiac (1) | Aphrodisiac (2)  Appetizing (1)  Tonic (1) | Antimicrobial and antioxidant [38] |
|  |  |  | Processed | Pickled | - | Eat |  | Aphrodisiac (1) |  |
| *Melissa officinalis* L. ^a^ | 3 | Upper stem (flowers and leaves) ^a^ | Processed | Shade dried | Aqueous infusion | Drink | Stomachache ^a^ (1)  Indigestion ^a^ (1) | Good for memory (1)  Sedative/calming agent ^a^ (1)  Good for sensitive stomach (1) | Herbal tea: antispasmodic in nervous and digestive disorders [39]  Antibacterial  [2] |
|  |  | Leaves | Processed | Shade dried | Aqueous infusion | Drink |  | Good for nervous system (1)  Good for stomach (1) |  |
| *Pyrus communis* L. ^b^ | 3 | Fruit | Fresh | - | Raw | Eat | D, D93 Styptic (1)  Spastic colitis (1) |  | Colon disorders [40]  Anti-diabetic, hypolipidemic [41] |
|  |  |  | Fresh or processed | Shade dried | Boil and mash (strain and keep the pulp) | Drink (consume diluted in water) | Spastic colitis (1) |  |  |
|  |  | Branch bark | Processed | Bark peel | Decoction | Drink | Cholesterol (1) |  |  |
| *Raphanus raphanistrum* L. ^a^ ^b^ | 3 | Whole aerial ^a^ | Fresh | - | Boil | Drink the decoction and eat the boiled plant material as salad |  | Antioxidant (1) | Antioxidant [42] |
| *Rubus sanctus* Schreb. ^a^ | 3 | Leaves ^a^ | Fresh or processed | Shade dried | Aqueous infusion | Drink | Diabetes (1)  Diarrhea ^a^ (1) | Rich in iron and polyphenols (1)  Antimicrobial (1) | Antinociceptive [43] |
| *Scolymus hispanicus* L. ^b^ | 3 | Whole aerial | Fresh | - | Boil or cook with food | Eat |  | Cleansing for the liver (1)  Antioxidant (1) | Antioxidant, high micronutrient content [44, 45] |
|  |  | Whole plant | Fresh | - | Boil | Drink the decoction and eat the boiled plant material as salad | Detoxifies and cleans the liver (1) | Good for liver health (1)  Rich in minerals (1) |  |
| *Sinapis arvensis* L. subsp. *arvensis* ^b^ | 3 | Whole aerial (sprouts) | Fresh | - | Boil | Drink the decoction and eat the boiled plant material as salad | Diabetes (1) | Antioxidant (1) | Antimicrobial, antioxidant [46] |
| *Urtica* sp. | 3 | Upper stem (flowers and leaves) | Fresh | - | Boil | Eat (salad) |  | Source of Iron (1)  Purifies the blood (1) | Beneficial to cure rheumatism, arthritis, allergies, antioxidant, prostrate problem (Benign prostatic hyperplasia), antidiabetic [47] |
|  |  |  |  | - | Raw | Skin application | Painful joints (1) |  |  |
|  |  |  |  | - | Decoction | Wash (hair) |  | Good for hair reconstruction (1) |  |
|  |  |  | Processed | Shade dried | Aqueous infusion | Drink |  | Source of Iron (1)  Purifies the blood (1) |  |
|  |  | Leaves | Fresh | - | Cook | Eat |  | Good for urinary system (1)  Prostate (1) |  |
| *Alkanna tinctoria* Tausch ^a^ | 2 | Root ^a^ | Processed | Lotion (Glass jar with the root's pill/skin + extra virgin olive oil. Store in Shade) | - | Skin application | Wound healing (2)  Insect bites/stinks (1) Hemorrhoids ^a^ (1) |  | Wound healing [48]  Antioxidant, wound healing, anti-inflammatory, antimicrobial [49] |
|  |  |  |  | Lotion (Glass jar with the root's pill/skin + coconut oil. Store in Shade) | - | Skin application |  | Skin revitalization (1) |  |
| *Anthriscus* sp. ^b^ | 2 | Whole aerial | Fresh | - | Raw or boil | Drink the decoction and eat the boiled plant material as salad | N/A | N/A |  |
| *Cistus* sp. | 2 | Leaves | Processed | Shade dried | Aqueous infusion | Drink | Stomachache (1)  Indigestion (1)  Gastroesophageal reflux disease (1) |  | Antibacterial, antimicrobial [50–52] |
|  |  | Upper stem (with leaves) | Processed | Shade dried | Aqueous infusion | Skin application | Cysts (1)  Eczema (1) |  |  |
|  |  |  |  |  |  | Drink |  | Cleans thee blood (1) |  |
| *Ficus carica* L. ^a^ ^b^ | 2 | Fruit ^a^ | Fresh | - | Raw (the milk that flows out of the fruit's top when you remove it from the tree) | Skin application | Skin warts (1) |  | Antioxidant [53]  Antispasmodic, antiplatelet (gut motility, inflammatory disorders) [54]  Antiwart [55] |
|  |  |  |  |  |  | Tooth application (Apply the milk - that flows out of the fruit's top when you remove it from the tree - on cotton swab and place it on the painful tooth) | Toothache (1) |  |  |
|  |  |  | Processed | Shade dried | - | Eat | Gastric ore intestinal pain (1) |  |  |
|  |  |  | Fresh or processed | Shade dried | Raw or processed | Eat |  | Rich in vitamins (1) |  |
| *Lactuca serriola* L. ^b^ | 2 | Whole aerial | Fresh | - | Boil | Eat | Cleans the liver (1) | Cleans the liver (1) | Spasmogenic, spasmolytic, bronchodilator, vasorelaxant  [56] |
| *Limonium sinuatum* (L.) Mill. ^b^ | 2 | Whole aerial or leaves | Fresh | - | Boil | Eat | N/A | N/A |  |
| *Papaver dubium* L. ^b^ | 2 | Whole aerial or leaves | Fresh | - | Boil | Eat | N/A | N/A |  |
| *Plantago lanceolata* L. ^b^ | 2 | Whole aerial | Fresh | - | Boil | Eat | Cleans the kidney/diuretic (1) |  | Antioxidant [57]  Antiinflammatory [58] |
| *Plantago weldenii* Rchb. ^b^ | 2 | Whole aerial | Fresh | - | Boil | Drink the decoction and eat the boiled plant material as salad |  | Healthy for gynecological system (1)  Good for kidney (1) |  |
| *Rumex obtusifolius* L. ^b^ | 2 | Whole aerial | Fresh | - | Boil or cook | Eat | Iron deficiency (1) | Rich in iron (1) | Antibacterial [59, 60] |
| *Silybum marianum* (L.) Gaertn. ^a^ ^b^ | 2 | Whole aerial ^a^ | Fresh | - | Boil | Drink the decoction and eat the boiled plant material as salad | Detoxifies and cleans the liver (1) | Good for liver health (1)  Rich in minerals (1) | Antioxidant, free radical scavenger, reduces adverse effect of chemical medicines such as anticancer [61, 62]  Hepatoprotective, anti-inflammatory, anticarcinogenic [62, 63] |
|  |  | Flowers | Processed | Shade dried | Aqueous infusion | Drink | Liver ^a^ and gall diseases (1) | Antioxidant for liver (1) |  |
|  |  | Seeds | Processed | Crush plant material and soak in grape vinegar for a night. Then you strain and add some honey (called Oksýmelo = Οξύμελο) | - | Drink | Liver and gall diseases (1) | Antioxidant for liver (1) |  |
| *Taraxacum* sp. *(**Stamnagáthi)* ^b^ | 2 | Whole aerial | Fresh | - | Raw, boil or cook | Drink the decoction and eat the boiled plant material as salad | N/A | N/A |  |
| *Acanthus spinosus* L. | 1 | Fruit | Fresh or processed | Shade dried | Raw or processed | Eat |  | Prostate (1) |  |
| *Amaranthus retroflexus* L. ^a^ ^b^ | 1 | Whole aerial ^a^ | Fresh | - | Boil | Eat |  | Rich in iron (1) |  |
| *Apium sp.* ^b^ | 1 | Whole aerial | Fresh or processed | Pickled | Boil or processed | Eat | Good for health issues related with iron and selenium deficiency (1) | Rich in iron and selenium (1) |  |
| *Ballota acetabulosa* (L.) Benth. | 1 | Upper stem (leaves) | Processed | Shade dried | Aqueous infusion | Drink | Diabetes (1) |  |  |
| *Calendula arvensis* M.Bieb*.* ^a^ | 1 | Flowers ^a^ | Processed | Lotion (Glass bottle with plant material + extra virgin olive oil. Store in Shade for a month. Then strain) | - | Skin application | Irritated skin / eczema (1) |  |  |
|  |  |  |  | Shade dried | Aqueous infusion | Drink | Irritated throat (1) |  |  |
| *Cardopatium corymbosum* (L.) Pers. | 1 | Root | Fresh | - | Grind | Skin application | Fungicide infections on hands (1) |  |  |
| *Carthamus dentatus* subsp. *ruber* (Link) Hanelt ^b^ | 1 | Whole aerial | Fresh | - | Boil | Eat | N/A | N/A |  |
| *Centaurium pulchellum* (Sw.) Druce | 1 | Upper stem (leaves) | Processed | Shade dried | Aqueous infusion | Drink | Diarrhea (1) |  |  |
| *Crataegus azarolus* L. | 1 | Branches, fruits, leaves | Processed | Tincture (Glass bottle: 150gr of plant material + 400gr Gin. Store for a month and then strain) | - | Drink | Heart arrythmias (1) |  |  |
| *Crepis zacintha* (L.) Babc. | 1 | Seeds | Fresh | - | Raw | Eat | Skin warts / Myrmecia (1) |  |  |
| *Cuscuta* sp. | 1 | Whole plant | Fresh or processed | Shade dried | Aqueous infusion | Drink | Hemorrhoids (1) |  |  |
| *Cydonia* sp. ^b^ | 1 | Fruit | Fresh | - | Raw | Eat | Diarrhea (1) |  |  |
| *Cynodon dactylon* (L.) Pers. | 1 | Whole plant | Fresh | - | Boil | Drink | Kidney stone disease (1) |  |  |
| *Dittrichia viscosa* (L.) Greuter | 1 | Leaves | Fresh | - | Crush | Skin application | Skin wounds / Haemostatic (1) |  |  |
| *Echium plantagineum* L. ^b^ | 1 | Whole aerial | Fresh | - | Boil | Eat | N/A | N/A |  |
| *Erodium cicutarium* (L.) L'Her. ^a^ ^b^ | 1 | Whole aerial | Fresh | - | Cook | Eat | N/A | N/A |  |
| *Galium aparine* L. | 1 | Upper stem | Processed | Crushed, Lotion (Glass bottle: crushed plant material + extra virgin olive oil. Store for 10 days and then strain) | - | Skin application | Painful joints (1) |  |  |
|  |  |  |  | Shade dried | Aqueous infusion | Drink | Painful joints (1) |  |  |
| *Hyoscyamus albus* L. ^a^ | 1 | Seeds ^a^ | Fresh | - | Throw the seeds on burning coal while holding a metal curved pan above it to block the smoke and make it ‘stick’ on it. Then turn/reverse the pan and add water in boiling temperature. | Eye application (Place the patient’s face above the pan having it covered with a towel – eyes should be wide open) | Eye parasites (1) |  |  |
| *Hypericum olympicum* L. | 1 | Upper stem (flowers and leaves) | Processed | Lotion (Transparent glass bottle full of plant material + extra virgin olive oil. Let in sun for min 40 days. Stir 1-2 times/day) | - | Skin application | Painful joints (1)  External hemorrhoids (1)  Skin wounds (1) |  |  |
| *Juglans regia* L. | 1 | Fruit | Fresh | - | Raw (peel) | Teeth application - teeth and gums |  | Teeth whitener (1) |  |
|  |  |  |  |  | Aqueous infusion (fruit peel) | Feet bath |  | Antifungal for feet (1) |  |
|  |  |  |  |  | Aqueous infusion (fruit peel) | Wash |  | Hair root strengthener (1) |  |
| *Laurus nobilis* L. ^b^ | 1 | Leaves | Processed | Shade dried | Cook | Eat | N/A | N/A |  |
| *Mentha aquatica* L. | 1 | Upper stem (flowers and leaves) | Processed | Shade dried | Aqueous infusion | Drink | Relieves from issues of the respiratory system (1)  Coughing (1) |  |  |
| *Mentha spicata* L. ^a^ | 1 | Leaves | Processed | Shade dried | Aqueous infusion | Drink | Indigestion (1) |  |  |
| *Morus sp.* | 1 | Fruit | Fresh or processed | Marmalade | - | Eat |  | Rich in iron (1)  Sedative / calming / sleep-inducing (1) |  |
| *Olea europaea* L.subsp. *oleaster* (Hoffmanns. & Link) Negodi | 1 | Fruit | Processed | Pickled | - | Eat | N/A | N/A |  |
| *Opuntia ficus-indica* (L.) Mill. ^b^ | 1 | Fruit | Fresh | - | Raw | Eat | N/A | N/A |  |
| *Prunus dulcis* (Mill.) D.A.Webb var. ^b^ | 1 | Fruit | Fresh | - | Raw | Eat | Gut worms (1) |  |  |
| *Quercus coccifera* L. | 1 | Trunk’s bark | Fresh | - | Grind and infuse | Mouth wash | Gingivitis (1) |  |  |
| *Robinia pseudoacacia* L. | 1 | Flowers | Processed | Shade dried | Aqueous infusion | Drink | Expectorant (1)  Stomach sedative (1) | Sedative / calming (1) |  |
| *Rosmarinus officinalis* L. ^a^ | 1 | Leaves ^a^ | Processed | Shade dried | Aqueous infusion | Drink |  | Good for memory (1) |  |
| *Rumex crispus* L. ^a^ ^b^ | 1 | Leaves | Fresh | - | Boil | Eat | N/A | N/A |  |
| *Salicornia europaea* L. ^b^ | 1 | Whole aerial | Fresh | - | Raw | Eat |  | Rich in minerals (1) |  |
| *Sambucus nigra* L. ^a^ | 1 | Flowers ^a^ | Processed | Shade dried | Aqueous infusion | Drink | Fever ^a^ (causes sweating) (1) |  |  |
| *Sinapis alba* L. ^b^ | 1 | Whole aerial | Fresh | - | Boil | Eat |  | Antioxidant (1) |  |
| *Solanum villosum* Mill. | 1 | Fruit | Fresh | - | Raw | Eat |  | Rich in vitamin C and lycopene (1)  Good for prostate health (1) |  |
| *Solanum nigrum* L. ^b^ | 1 | Upper stem (with leaves) | Fresh | - | Boil | Eat | N/A | N/A |  |
| *Dioscorea communis* (L.) Caddick & Wilkin ^b^ | 1 | Upper stem (with leaves) | Fresh | - | Boil or cook | Drink the decoction and eat the boiled (salad) or cooked (omelet or fricassee) plant material | N/A | N/A |  |
| *Viola kitaibeliana* Schult. | 1 | Flowers | Processed | Shade dried | Aqueous infusion | Drink | Expectorant (1) | Sedative/calming agent (1) |  |
| *Vitex agnus-castus* L. | 1 | Flowers | Processed | Shade dried | Aqueous infusion | Drink | N/A | N/A |  |

Abbreviations: f=frequency of plant referrals in free-listing exercise

^a^ Plant species, corresponding plant parts and medicinal uses presented in the study of Axiotis et al. [64].

^b^ Plant taxa used primarily as food or flavor enhancer rather than medicine.

References

1. Faleiro L, Miguel G, Gomes S, Costa L, Venâncio F, Teixeira A, et al. Antibacterial and Antioxidant Activities of Essential Oils Isolated from Thymbra capitata L. (Cav.) and Origanum vulgare L. J Agr Food Chem. 2005;53:8162–8.

2. Reichling J, Schnitzler P, Suschke U, Saller R. Essential oils of aromatic plants with antibacterial, antifungal, antiviral, and cytotoxic properties–an overview. J Complement Med Res. 2009;16:79–90.

3. El Moussaoui N, Sanchez G, Idaomar M, Mansour AI, Abrini J, Aznar R. Antibacterial and antiviral activities of essential oils of Northern Moroccan plants. Biotechnol J Int. 2013:318–31.

4. Zhang X-L, Guo Y-S, Wang C-H, Li G-Q, Xu J-J, Chung HY, et al. Phenolic compounds from Origanum vulgare and their antioxidant and antiviral activities. Food Chem. 2014;152:300–6.

5. Radulović N, Stankov-Jovanović V, Stojanović G, Šmelcerović A, Spiteller M, Asakawa Y. Screening of in vitro antimicrobial and antioxidant activity of nine Hypericum species from the Balkans. Food Chem. 2007;103:15–21.

6. Barnes J, Anderson LA, Phillipson JD. St John's wort (Hypericum perforatum L.): a review of its chemistry, pharmacology and clinical properties. J Pharm Pharmacol. 2001;53:583–600.

7. Miller AL. St. John's Wort (Hypericum perforatum): clinical effects on depression and other conditions. Altern Med Rev. 1998;3:18–26.

8. Gaster B, Holroyd J. St John's Wort for Depression: A Systematic Review. Arch Intern Med. 2000;160:152–6.

9. Rego J-C, Benkiki N, Chosson E, Kabouche Z, Seguin E, Costentin J. Antidepressant-like effect of hyperfoliatin, a polyisoprenylated phloroglucinol derivative from Hypericum perfoliatum (Clusiaceae) is associated with an inhibition of neuronal monoamines uptake. Eur J Pharmacol. 2007;569:197–203.

10. Süntar IP, Akkol EK, Yılmazer D, Baykal T, Kırmızıbekmez H, Alper M, Yeşilada E. Investigations on the in vivo wound healing potential of Hypericum perforatum L. J Ethnopharm. 2010;127:468–77.

11. Samadi S, Khadivzadeh T, Emami A, Moosavi NS, Tafaghodi M, Behnam HR. The Effect of Hypericum perforatum on the Wound Healing and Scar of Cesarean. J Altern Complement Med. 2010;16:113–7.

12. Jamalian A, Shams-Ghahfarokhi M, Jaimand K, Pashootan N, Amani A, Razzaghi-Abyaneh M. Chemical composition and antifungal activity of Matricaria recutita flower essential oil against medically important dermatophytes and soil-borne pathogens. J Mycol Med. 2012;22:308–15.

13. Pereira RP, Fachinetto R, Souza Prestes A de, Puntel RL, Santos da Silva, Gloria Narjara, Heinzmann BM, et al. Antioxidant Effects of Different Extracts from Melissa officinalis, Matricaria recutita and Cymbopogon citratus. Neurochem Res. 2009;34:973–83.

14. Ait-Ouazzou A, Lorán S, Arakrak A, Laglaoui A, Rota C, Herrera A, et al. Evaluation of the chemical composition and antimicrobial activity of Mentha pulegium, Juniperus phoenicea, and Cyperus longus essential oils from Morocco. Food Res Int. 2012;45:313–9.

15. Razavi SM, Zarrini G, Molavi G, Ghasemi G. Bioactivity of malva sylvestris L., a medicinal plant from iran. Iran J Basic Med Sci. 2011;14:574–9.

16. Watanabe E, Tanomaru JMG, Nascimento AP, Matoba-Júnior F, Tanomaru-Filho M, Yoko Ito I. Determination of the maximum inhibitory dilution of cetylpyridinium chloride-based mouthwashes against Staphylococcus aureus: an in vitro study. J Appl Oral Sci. 2008;16:275–9.

17. Fu Z, Wang H, Hu X, Sun Z, Han C. The pharmacological properties of Salvia essential oils. J Appl Pharm Sci. 2013;3:122.

18. Clare BA, Conroy RS, Spelman K. The Diuretic Effect in Human Subjects of an Extract of Taraxacum officinale Folium over a Single Day. J Altern Complement Med. 2009;15:929–34.

19. Senatore F, Napolitano F, Ozcan M. Composition and antibacterial activity of the essential oil from Crithmum maritimum L. (Apiaceae) growing wild in Turkey. Flavour Fragr J. 2000;15:186–9.

20. Meot-Duros L, Magné C. Antioxidant activity and phenol content of Crithmum maritimum L. leaves. Plant Physiol Biochem. 2009;47:37–41.

21. Uddin MK, Juraimi A, Hossain M, Nahar A, Ali M, Rahman M. Purslane weed (Portulaca oleracea): A prospective plant source of nutrition, omega-3 fatty acid, and antioxidant attributes. Sci World J 2014.

22. Young-Ock K, Sang-Won L, Sae W, Park H, Eun S. Anti-inflammatory effects of Portulaca oleracea L. on the LPS-induced RAW 264.7 cells. J Med Plant Res. 2015;9:407–11.

23. Teugwa CM, Mejiato PC, Zofou D, Tchinda BT, Boyom FF. Antioxidant and antidiabetic profiles of two African medicinal plants: Picralima nitida (Apocynaceae) and Sonchus oleraceus (Asteraceae). BMC Complement Altern Med. 2013;13:175.

24. Torres-González L, Cienfuegos-Pecina E, Perales-Quintana MM, Alarcon-Galvan G, Muñoz-Espinosa LE, Pérez-Rodríguez E, Cordero-Pérez P. Nephroprotective Effect of Sonchus oleraceus Extract against Kidney Injury Induced by Ischemia-Reperfusion in Wistar Rats. Oxid Med Cell Longev. 2018;2018:7.

25. Lattanzio F, Greco E, Carretta D, Cervellati R, Govoni P, Speroni E. In vivo anti-inflammatory effect of Rosa canina L. extract. J Ethnopharm. 2011;137:880–5.

26. Daels-Rakotoarison DA, Gressier B, Trotin F, Brunet C, Luyckx M, Dine T, et al. Effects of Rosa canina fruit extract on neutrophil respiratory burst. Phytother Res. 2002;16:157–61.

27. Fan C, Pacier C, Martirosyan D. Rosehip(Rosa caninaL): A functional food perspective. Functional Foods in Health and Disease. 2014;volume 4:Page 493-509.

28. Kofinas C, Chinou J, Harvala A, Gally A. Composition and Antibacterial Activity of the Essential Oil of Tordylium apulum L. J Essent Oil Res. 1993;5:33–6.

29. Simopoulos AP. Omega-3 fatty acids and antioxidants in edible wild plants. Biol Res. 2004;37:263–77.

30. Di Maro A, Pacifico S, Fiorentino A, Galasso S, Gallicchio M, Guida V, et al. Raviscanina wild asparagus (Asparagus acutifolius L.): A nutritionally valuable crop with antioxidant and antiproliferative properties. Food Res Int. 2013;53:180–8.

31. Zarei L, Pourjabali M, Naghdi N, Naji Haddadi S, Bahmani E. A systematic review of the most important medicinal plants native to Iran effective on testicular morphology and hormonal testicular function. J Pharm Sci Res. 2017;9:562–7.

32. Rahimi R, Ardekani MRS. Medicinal properties of Foeniculum vulgare Mill. in traditional Iranian medicine and modern phytotherapy. Chin J Integr Med. 2013;19:73–9.

33. Rouis Z, Abid N, Koudja S, Yangui T, Elaissi A, Cioni PL, et al. Evaluation of the cytotoxic effect and antibacterial, antifungal, and antiviral activities of Hypericum triquetrifolium Turra essential oils from Tunisia. BMC Complement Altern Med. 2013;13:24.

34. Kızıl G, Toker Z, Özen HÇ, Aytekin Ç. The antimicrobial activity of essential oils of Hypericum scabrum, Hypericum scabroides and Hypericum triquetrifolium. Phytother Res. 2004;18:339–41.

35. Zaman R, Basar SN. A review article of Beekhe Kasni (Cichorium intybus) its traditional uses and pharmacological actions. Res J Pharm Sci. 2013;2319:555X.

36. Shaikh T, Mujum A, Wasimuzzama K, Rub RA. An overview on phytochemical and pharmacological profile of Cichorium intybus Linn. Br J Pharmacol. 2010;2:298–307.

37. Aboul-Enein AM, El-Ela FA, Shalaby EA, El-Shemy HA. Traditional medicinal plants research in Egypt: studies of antioxidant and anticancer activities. J Med Plant Res. 2012;6:689–703.

38. Omri Hichri A, Mosbah H, Majouli K, Besbes Hlila M, Ben Jannet H, Flamini G, et al. Chemical composition and biological activities of Eruca vesicaria subsp. longirostris essential oils. Pharm Biol. 2016;54:2236–43.

39. Carnat AP, Carnat A, Fraisse D, Lamaison JL. The aromatic and polyphenolic composition of lemon balm (Melissa officinalis L. subsp. officinalis) tea. Pharm Acta Helv. 1998;72:301–5.

40. Ilhan M, Akkol EK, Taştan H, Dereli FTG, Tümen I. Efficacy of Pyrus elaeagnifolia subsp. elaeagnifolia in acetic acid–induced colitis model. Open Chem. 2019;17:13–22.

41. Velmurugan C, Bhargava A. Anti-diabetic and hypolipidemic activity of fruits of Pyrus communis L. in hyperglycemic rats. Asian J Pharm Clin Res. 2013;6:108–11.

42. Küçükboyaci N, Güvenç A, Turan NN, Aydin A. Antioxidant activity and total phenolic content of aqueous extract from Raphanus Raphanistrum L. Turk J Pharm Sci. 2012;9:93–100.

43. Erdemoglu N, Küpeli E, Yeşilada E. Anti-inflammatory and antinociceptive activity assessment of plants used as remedy in Turkish folk medicine. J Ethnopharm. 2003;89:123–9.

44. Vardavas CI, Majchrzak D, Wagner K-H, Elmadfa I, Kafatos A. The antioxidant and phylloquinone content of wildly grown greens in Crete. Food Chem. 2006;99:813–21.

45. Morales P, Ferreira ICFR, Carvalho AM, Sánchez-Mata MC, Cámara M, Fernández-Ruiz V, et al. Mediterranean non-cultivated vegetables as dietary sources of compounds with antioxidant and biological activity. LWT-Food Sci Technol. 2014;55:389–96.

46. Rad JS, Alfatemi MH, Rad MS, Sen DJ. Phytochemical and antimicrobial evaluation of the essential oils and antioxidant activity of aqueous extracts from flower and stem of Sinapis arvensis L. Am J Adv Drug Deliv. 2013;1:1–10.

47. Pant V, Sundriyal RC. Nutritional and therapeutic efficacy of Stinging Nettle-A review. J Ethnobiol Trad Med. 2016;126:1240–54.

48. Papageorgiou VP. Wound healing properties of naphthaquinone pigments from Alkanna tinctoria. Experientia. 1978;34:1499–501.

49. Assimopoulou AN, Boskou D, Papageorgiou VP. Antioxidant activities of alkannin, shikonin and Alkanna tinctoria root extracts in oil substrates. Food Chem. 2004;87:433–8.

50. Demetzos C, Loukis A, Spiliotis V, Zoakis N, Stratigakis N, Katerinopoulos HE. Composition and Antimicrobial Activity of the Essential oil of Cistus creticus L. J Essent Oil Res. 1995;7:407–10.

51. Demetzos CN, Chinou JB, Charvala CE, Homatidou VI. The essential oil of Cistus parviflorus and its antimicrobial activity in comparison with C. monspeliensis. Fitoterapia. 1990;61:439–42.

52. Demetzos C, Angelopoulou D, Perdetzoglou D. A comparative study of the essential oils of Cistus salviifolius in several populations of Crete (Greece). Biochem Syst Ecol. 2002;30:651–65.

53. Solomon A, Golubowicz S, Yablowicz Z, Grossman S, Bergman M, Gottlieb HE, et al. Antioxidant activities and anthocyanin content of fresh fruits of common fig (Ficus carica L.). J Agr Food Chem. 2006;54:7717–23.

54. Gilani AH, Mehmood MH, Janbaz KH, Khan A-u, Saeed SA. Ethnopharmacological studies on antispasmodic and antiplatelet activities of Ficus carica. J Ethnopharm. 2008;119:1–5.

55. Bohlooli S, Mohebipoor A, Mohammadi S, Kouhnavard M, Pashapoor S. Comparative study of fig tree efficacy in the treatment of common warts (Verruca vulgaris) vs cryotherapy. Int J Dermatol. 2007;46:524–6.

56. Janbaz KH, Latif MF, Saqib F, Imran I, Zia-Ul-Haq M, Feo V de. Pharmacological Effects of Lactuca serriola L. in Experimental Model of Gastrointestinal, Respiratory, and Vascular Ailments. Evid Based Complement Alternat Med. 2013;2013:304394.

57. Dalar A, Türker M, Konczak I. Antioxidant capacity and phenolic constituents of Malva neglecta Wallr. and Plantago lanceolata L. from Eastern Anatolia Region of Turkey. J Herb Med. 2012;2:42–51.

58. Marchesan M, Paper DH, Hose S, Franz G. Investigation of the antiinflammatory activity of liquid extracts of Plantago lanceolata L. Phytother Res. 1998;12:S33-S34.

59. Harshaw D, Nahar L, Vadla B, Saif-E-Naser G, Sarker S. Bioactivity of Rumex obtusifolius (Polygonaceae). Arch biol sci. 2010;62:387–92.

60. Ginovyan M, Petrosyan M, Trchounian A. Antimicrobial activity of some plant materials used in Armenian traditional medicine. BMC Complement Altern Med. 2017;17:50.

61. Bahmani M, Shirzad H, Rafieian S, Rafieian-Kopaei M. Silybum marianum: Beyond Hepatoprotection. J Evid Based Complementary Altern Med. 2015;20:292–301.

62. Shaker E, Mahmoud H, Mnaa S. Silymarin, the antioxidant component and Silybum marianum extracts prevent liver damage. Food Chem Toxicol. 2010;48:803–6.

63. Fraschini F, Demartini G, Esposti D. Pharmacology of Silymarin. Clin Drug Investig. 2002;22:51–65.

64. Axiotis E, Halabalaki M, Skaltsounis LA. An Ethnobotanical Study of Medicinal Plants in the Greek Islands of North Aegean Region. Front Pharmacol. 2018;9:409.
