# Supplementary material for: Local knowledge about sustainable harvesting and availability of wild medicinal plant species in Lemnos island, Greece
Source: J Ethnobiol Ethnomed. 2020 Jun 19;16:36. doi: 10.1186/s13002-020-00390-4 (PMC7304145; doi:10.1186/s13002-020-00390-4)
Supplement: Supplementary file 3 — Additional file 3. Susceptibility assessment of the most frequently collected wild medicinal plants taxa (f>3) to overcollection (n=16). [file 13002_2020_390_MOESM3_ESM.docx]

Additional file 3: Susceptibility assessment of the most frequently collected wild medicinal plant taxa (f>3) to overcollection (n=16).

| **Scientific name** | **f** | **Life history traits [1]** | **Plant part harvested** | **Susceptibility of species to overcollection [2, 3]** |
| --- | --- | --- | --- | --- |
| *Thymbra capitata* (L.) Cav. | 15 | Perennial | 46% Flower, 54% Upper stem | Low |
| *Origanum vulgare* L. subsp. *hirtum* (Link) letsw. | 12 | Perennial | Upper stem | Low |
| *Hypericum perfoliatum* L. and *Hypericum perforatum* L. | 11 | Perennial and Perennial | 22% Flower, 78% Upper stem | Low |
| *Matricaria chamomilla* L. | 10 | Annual | Flower | Medium |
| *Mentha pulegium* L. | 10 | Perennial | Upper stem | Low |
| *Malva sylvestris* L. | 8 | Perennial (Biennial) | 83% Flower, 17% Upper stem | Low |
| *Salvia spp.* | 7 | Perennial | 57% Upper stem, 43% Flower | Low |
| *Taraxacum* spp. | 7 | Perennial | Whole aerial | Low |
| *Crithmum maritimum* L. | 6 | Perennial | 38% Leaves, 62% Upper stem | Low |
| *Portulaca oleracea* L. | 6 | Annual | 20% Leaves, 80% Upper stem | Low |
| *Sonchus oleraceus (*L.) L. | 6 | Biennial | Whole aerial  (young/fresh leaves) | Medium |
| *Rosa canina* L. | 5 | Perennial | 29% Flower, 71% Fruit | Low |
| *Tordylium apulum* L. | 5 | Perennial | Whole aerial  (young/fresh leaves) | Low |
| *Asparagus acutifolius* L. | 4 | Perennial | 40% Whοle aerial, 60% Upper stem | Low |
| *Capparis spinosa* L. | 4 | Perennial | Upper stem - including flower buds, fruit and leaves | Low |
| *Foeniculum vulgare* Mill | 4 | Perennial | 20% Upper stem, 10% Flower, 40% Leaves, 30% Seed | Low |
| *Hypericum triquetrifolium* Turra | 4 | Perennial | 25% Flower, 75% Upper stem | Low |

Abbreviations: f=frequency of referrals for plants that had been harvested by the informants (n=16) at least once within the last four years.

References

1. Plants For A Future. Plants for a future. 2019. https://www.pfaf.org/user/Default.aspx. Accessed 05.2019.

2. Schippmann UWE, Leaman D, Cunningham AB. A comparison of cultivation and wild collection of medicinal and aromatic plants under sustainability aspects. Frontis. 2006:75–95.

3. Shackleton CM, Pandey AK, Ticktin T, editors. Ecological sustainability for non-timber forest products: dynamics and case studies of harvesting. Milton Park, Abingdo: Routledge; 2015.
